# Differential cellular proliferation underlies heterochronic generation of cranial diversity in phyllostomid bats

**DOI:** 10.1186/s13227-020-00156-9

**Published:** 2020-06-02

**Authors:** Jasmin Camacho, Rachel Moon, Samantha K. Smith, Jacky D. Lin, Charles Randolph, John J. Rasweiler, Richard R. Behringer, Arhat Abzhanov

**Affiliations:** 1 Department of Organismic and Evolutionary Biology, Harvard University, Cambridge, MA 02138 USA; 2 Department of Genetics, Blavatnik Institute, Harvard Medical School, Boston, MA 02115 USA; 3 Department of Obstetrics and Gynecology, State University Downstate Medical Center, Brooklyn, USA; 4 Department of Genetics, University of Texas MD Anderson Cancer Center, Houston, USA; 5 Department of Life Sciences, Imperial College London, Silwood Park Campus Buckhurst Road, Ascot, Berkshire, SL5 7PY UK; 6 Natural History Museum, Cromwell Road, London, SW7 5BD UK

**Keywords:** Development, Evolution, Heterochrony, Cell proliferation, Craniofacial, Morphology, Bat

## Abstract

**Background:**

Skull diversity in the neotropical leaf-nosed bats (Phyllostomidae) evolved through a heterochronic process called peramorphosis, with underlying causes varying by subfamily. The nectar-eating (subfamily Glossophaginae) and blood-eating (subfamily Desmondontinae) groups originate from insect-eating ancestors and generate their uniquely shaped faces and skulls by extending the ancestral ontogenetic program, appending new developmental stages and demonstrating peramorphosis by hypermorphosis. However, the fruit-eating phyllostomids (subfamilies Carollinae and Stenodermatinae) adjust their craniofacial development by speeding up certain developmental processes, displaying peramorphosis by acceleration. We hypothesized that these two forms of peramorphosis detected by our morphometric studies could be explained by differential growth and investigated cell proliferation during craniofacial morphogenesis.

**Results:**

We obtained cranial tissues from four wild-caught bat species representing a range of facial diversity and labeled mitotic cells using immunohistochemistry. During craniofacial development, all bats display a conserved spatiotemporal distribution of proliferative cells with distinguishable zones of elevated mitosis. These areas were identified as modules by the spatial distribution analysis. Ancestral state reconstruction of proliferation rates and patterns in the facial module between species provided support, and a degree of explanation, for the developmental mechanisms underlying the two models of peramorphosis. In the long-faced species, *Glossophaga soricina*, whose facial shape evolved by hypermorphosis, cell proliferation rate is maintained at lower levels and for a longer period of time compared to the outgroup species *Miniopterus natalensis*. In both species of studied short-faced fruit bats, *Carollia perspicillata* and *Artibeus jamaicensis*, which evolved under the acceleration model, cell proliferation rate is increased compared to the outgroup.

**Conclusions:**

This is the first study which links differential cellular proliferation and developmental modularity with heterochronic developmental changes, leading to the evolution of adaptive cranial diversity in an important group of mammals.
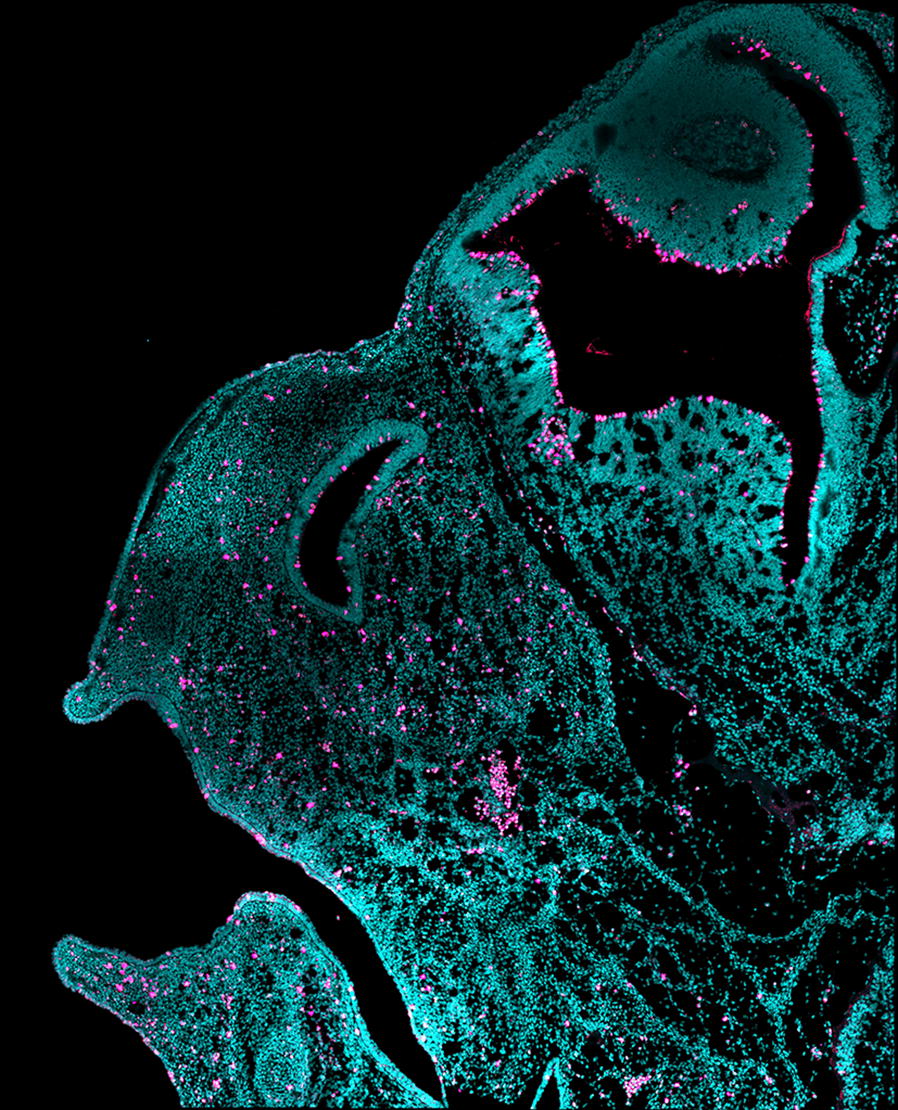

## Introduction

**Fig. 1:**
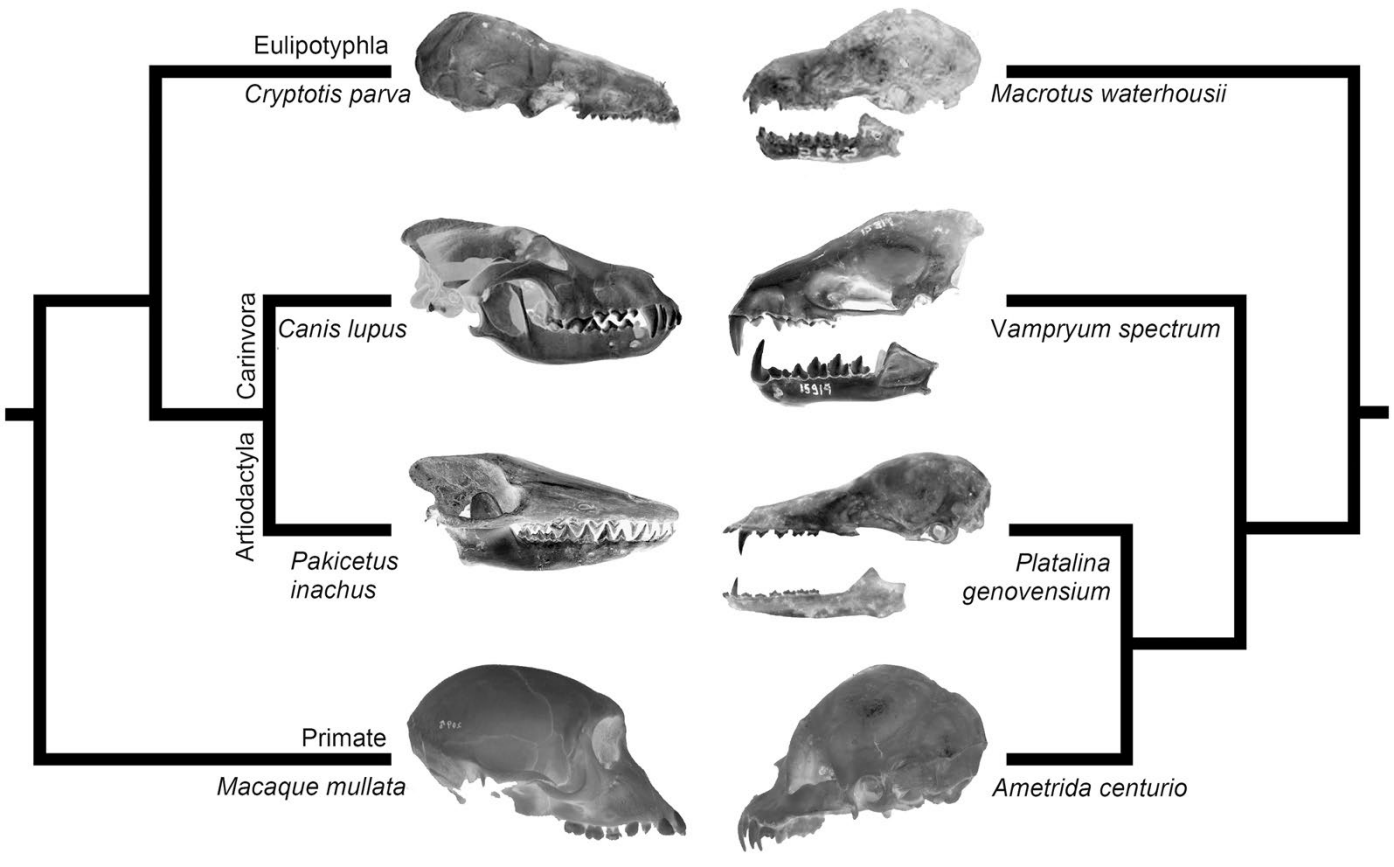
Order-level changes in mammals are reflected in closely related bat species. Cranial diversity in eutherian mammals (left) is mirrored in phyllostomid evolution (right). Variation in morphology is represented by the shrew *Cryptotis parva* (UTEP 1345) and *Macrotus waterhousii* (CMNA 13450), by carnivores *Canis lupus* (TMM 1709) and *Vampyrum spectrum* (RMNH 15914), by the long-faced whale-ancestor *Pakicetus inachus* (NHML) and long-faced nectar bat *Platalina genovensium* (CEBIOMAS 224), and by the short-faced primate *Macaque mullata* (DKY 0209) and short-faced fruit bat *Ametrida centurio* (UMMZ 53108). The simplified eutherian phylogeny is based on [70] and display members of the Orders Eulipotyphla, Carnivora, Artiodactyla, and Primate. The simplified phyllostomid phylogeny is based on [15]. All images are under a Creative Commons license. CEBIOMAS:  Centro de Biotecnologia da Mata Atlântica; CMNA: Colección Nacional de Mamíferos; DKY: Dokkyo Medical University; NHML: Natural History Museum, London; RMNH: Rijksmuseum van Natuurlijke Historie; TMM: University of Texas; UMMZ: University of Michigan Museum of Zoology; UTEP: The University of Texas at El Paso

Neotropical leaf-nosed bats (family Phyllostomidae, superfamily Noctilionoidea) evolved fantastic cranial variation associated with adaptations into diverse dietary niches. Among bats, phyllostomids have the largest array of distinct feeding strategies: sanguivory, insectivory, frugivory, nectarivory, carnivory, and omnivory [6, 7, 20]. The diversity of phyllostomid skulls encompasses many skull phenotypes [18] found in phylogenetically distant mammalian orders, such as carnivores and primates [17]. Therefore, this clade of closely related species of mammals offers a unique opportunity to gain key new insights into the origins of the order-level evolutionary diversity in Class Mammalia that have arisen over tens of millions of years (Fig. 1). In mammals, heterochrony, a change in the exact timing of developmental events relative to the ancestor, and, in particular, a change to the order and timing of cranial bone ossifications (known as “sequence heterochrony”), has already been shown as a major source of skull diversification [24, 25, 28, 45]. These modifications can result in descendants either resembling juveniles of the ancestor (paedomorphosis) or have gone beyond to become a more derived version of the ancestor (peramorphosis) [3]. Paedomorphosis is quite well known from the popular example of the axolotl salamander, which retains the larval gills and aquatic lifestyle found in juveniles of close relatives [26, 68]. The “Irish elk”, an extinct deer species with enormous exaggerated antlers, is a textbook example of peramorphosis [26]. Our recent morphometric studies on adult, juvenile and embryonic skulls demonstrated that several skull shapes within phyllostomid cranial diversity evolved by peramorphosis [11]. Long-faced nectar-eating (subfamily Glossophaginae) and short-faced blood-eating (subfamily Desmondontinae*)* bats generate ecomorph-specific skulls by extending the ancestral ontogenetic program and appending new late developmental stages, thus demonstrating peramorphosis by *hypermorphosis* [3]. Short-faced fruit-eating bats (subfamilies Carollinae and Stenodermatinae) adjust their craniofacial development by speeding up certain developmental processes, displaying peramorphosis by *acceleration* [3]. However, while our morphometric analyses implicated heterochronic modifications to the developmental programs during phyllostomid evolution, the precise cellular and molecular mechanisms behind these developmental changes remained unknown. In fact, there are very few studies, especially in vertebrates, which dissect the cellular and molecular mechanisms behind heterochrony.

To better understand the mechanistic nature of heterochrony-driven morphological evolution in phyllostomids, we aimed to investigate cell behavior during their craniofacial development and determine how alterations in cellular biology affect cranial shapes in different species. Once morphogenesis is understood at the cellular level, we can begin to explain how diversity is generated by changes in the underlying developmental processes [29]. Knowledge of the alterations at the cellular level, in turn, creates a platform allowing further dissection at the molecular and genetic levels.

The most important proximal process underlying morphogenesis is species-specific differential growth via cellular proliferation [1, 2, 9, 33, 35, 38, 75, 76]. Cell proliferation depends on several factors, such as the number of available precursor cells, the length of the period of mitosis, and the duration of the cell cycle [50]. Recent improvements in high-throughput, high-resolution imaging [19, 22] and in imaging analysis [65, 77] allow cells from a wide range of tissues and species to be studied in great detail. Thus, comparative analyses on differences in cellular behaviors across species, interpreted in an appropriate phylogenetic framework, can yield enhanced metrics for better characterization of morphological evolution.

**Fig. 2:**
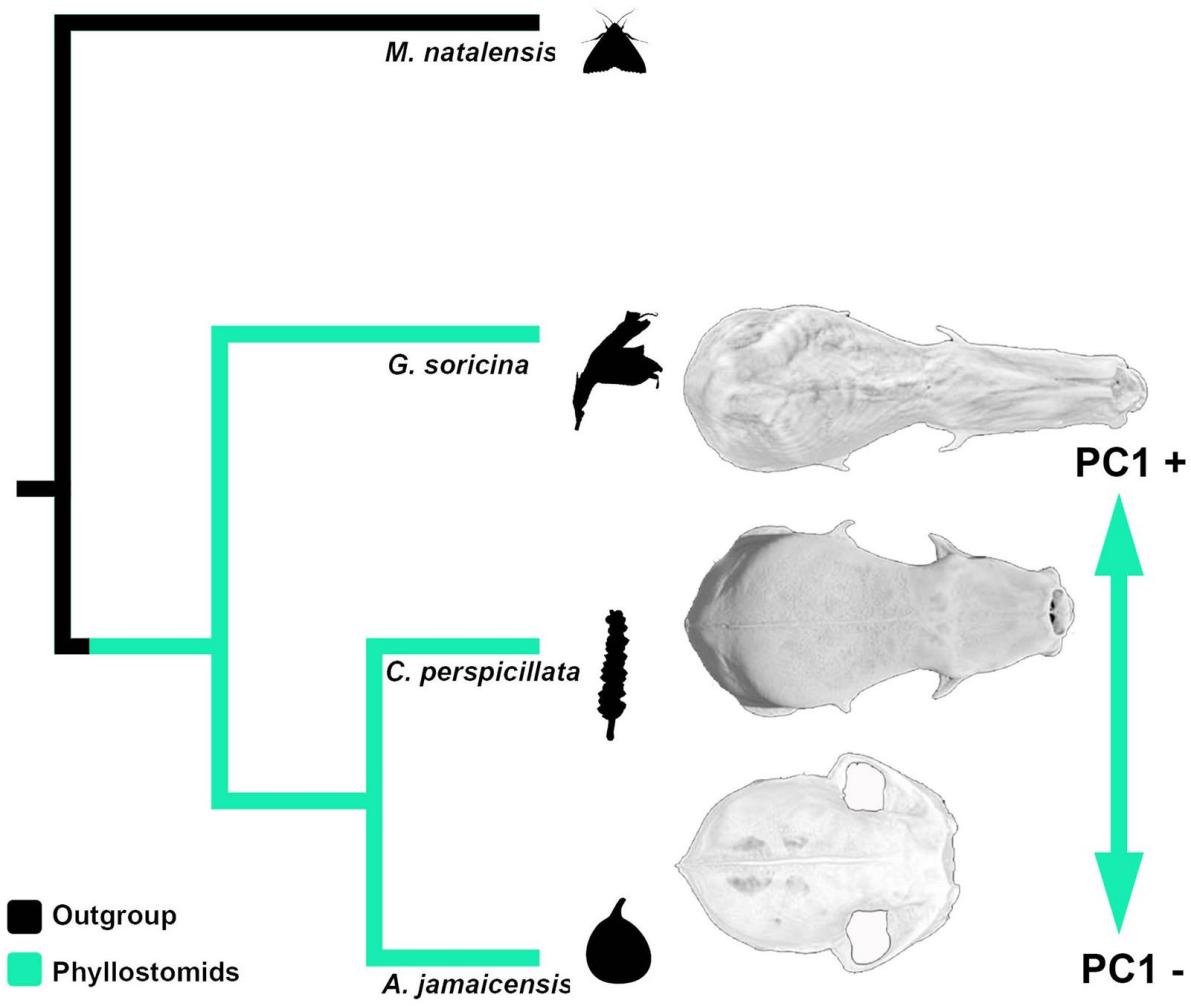
Comparisons within a phylogenetic context. Species different in their diets and craniofacial length morphology were compared within a phylogenetic context. A silhouette of the primary source of food for each species is represented at each terminal node for *M. natalensis* (moth), *G. soricina* (neotropical bell-flower), *C. perspicillata* (piper fruit), and *A. jamaicensis* (fig). Dorsal views of µCT images capturing PC1 diversity from our prior principal component (PC) analysis [11] on skull shape show an ‘average’ or generalist shape in *Carollia perspicillata*, an extreme PC1-positive score in *Platalina genovensium* (nectarivore; MCZ-32948), and an extreme PC1-negative score in *Centurio senex* (frugivore; AMNH-M175651). AMNH: American Museum of Natural History; MCZ: Museum of Comparative Zoology at Harvard

**Fig. 3:**
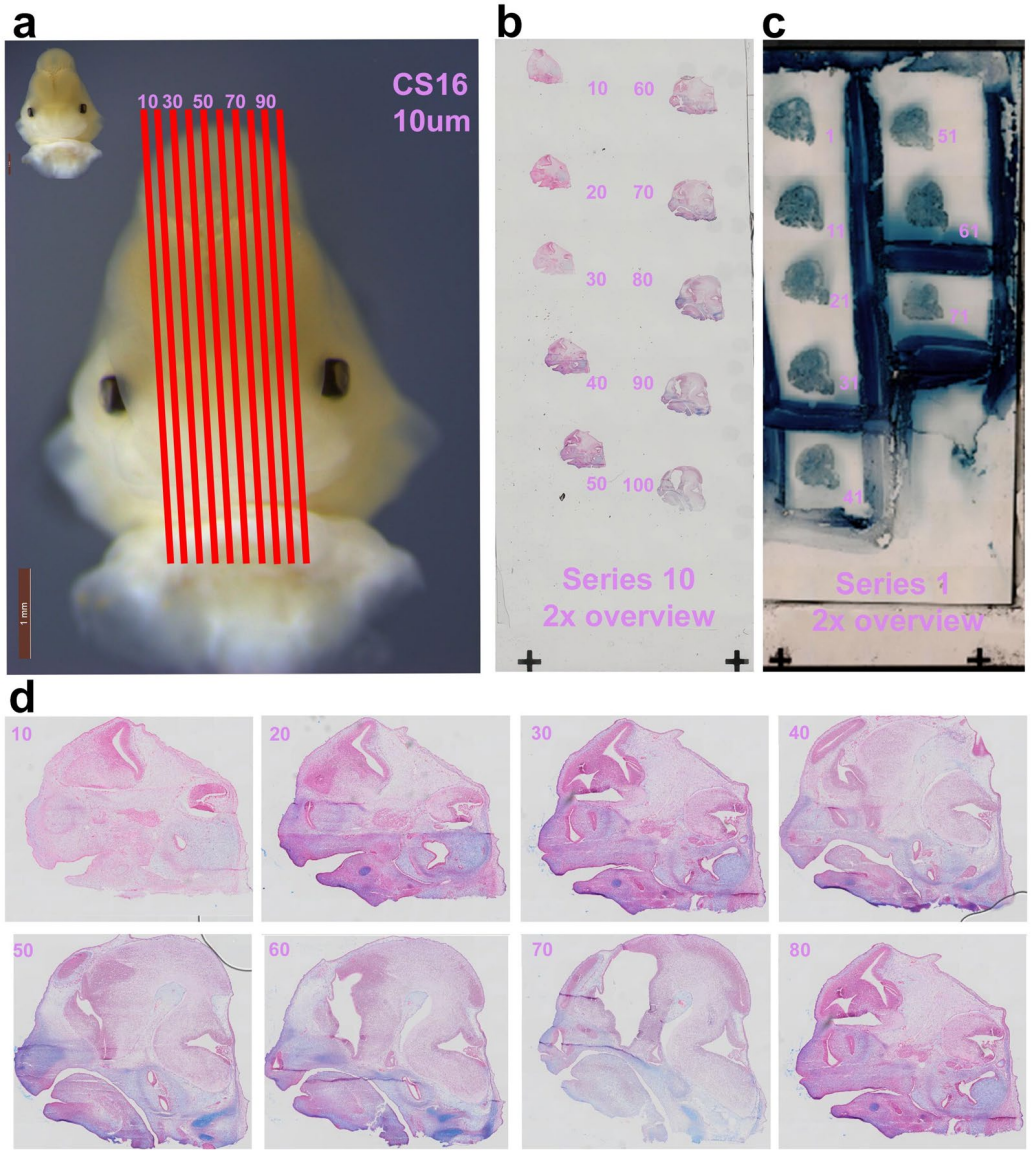
Serial sectioning for multiple experiments. Sagittal sections spanning midfacial tissue between the eyes were analyzed. Sagittal sections along the medial–lateral axes are shown for *C. perspicillata* at CS16 (**a**). Shown is slide series 10 stained with H&E and counterstained with alcian blue (**b**) for anatomical reference. Slide series 1 is used for immunohistochemistry (**c**) to target proliferating cells. Sagittal sections imaged at 20× magnification are positioned along the medial–lateral axis to visualize developing cranial tissue. H&E staining highlights the cytoplasm of cells in pink (eosin); cartilage cells in blue (alcian); and nuclei are stained black (hematoxylin) (**d**). Scale bar: 1 mm

Here, we investigate cellular proliferation underlying distinct morphogenetic trajectories in facial development caused by peramorphosis in phyllostomid bats. Our previous geometric morphometric analysis of phyllostomid skull shape showed that the most significant axis of morphologic change was the length of the skull and snout [11], which was captured by principal component (PC) 1 (Fig. 2). Thus, we focus on phyllostomid species, which feature significant differences in overall cranial length [15, 31] that we could collect from the wild: *Carollia perspicillata*, a predominantly frugivorous bat [56, 78] with a face near the center of cranial shape morphospace (Fig. 2); *Artibeus jamaicensis*, a predominantly frugivorous bat [41] with a short and wide face; and *Glossophaga soricina*, a predominantly nectarivorous and pollenivorous bat [12] with an elongated head and narrow face. These dietary specialists were compared to *Miniopterus natalensis* (family Miniopteridae), a representative insect-feeding outgroup species with a relatively unmodified face from South Africa, the geographic area of origin for Neotropical species in Noctilionoidea [27, 44, 64]. Embryos from each species were collected during stages CS16, CS17, and CS18 (approximately 50, 54, and 60 days of gestation, respectively) undergoing craniofacial elongation [13]. Serial sagittal sections from each embryo head were used for immunohistochemistry (Fig. 3) to detect a known mitotic marker, the Ser10-phosphorylated histone H3 or PH3. We hypothesized that if the alteration in developmental timing occurred at the cellular level, we would expect a localized temporal change in cell proliferation when compared to the ancestral mode of development. More specifically, compared to the ancestor, we anticipated a higher rate of proliferation in fruit bats and a more extended period of cell proliferation in nectar bats associated with acceleration and hypermorphosis, respectively, as we previously detected in these taxa morphologically [11].

## Results

### Craniofacial development in bat embryos

We collected a complete range of embryo ages for only one species, *C. perspicillata,* and we staged them with the Carnegie stage (CS) table (Additional file 1: Table S1) as CS16–CS24. For *C. perspicillata,* an emerging model for bat craniofacial development, we first summarize the main sequence of morphological changes (Table 1) and compare this to what is known for mouse embryonic development at comparable stages. We find that features of limb development normally used for stage-matching embryos in bats and mice [13, 32, 55, 67, 72, 73] reveal a global temporal shift in craniofacial development among these two groups. Thus, at equivalent developmental stages, based on limb morphology, the chiropteran head matures more quickly than in the mouse (Additional file 2: Figure S1).

**Table 1:** Carnegie stages and gestation age in the model bat *C. perspicillata*

Carnegie stage (CS)	Bat day of gestation	Mouse equivalent limbs	Mouse equivalent craniofacial	Key events
CS16	E50	E12.5	E14.5	Craniofacial prominences completely fused Undifferentiated mesenchyme in medial midface Meckel’s cartilage as circular condensation Posterior cranial base cartilage begins to organize
CS17	E54	E13.0	E15.0	Thickening of presumptive leaf-nose Lateral nasal capsule cartilage thickens Mesenchymal thickenings of olfactory turbinates Tooth development similar to mouse (thickening)
CS18	E60	E13.5	E15.5	Dorsal ventral expansion at the resting zone of basisphenoid Resting zone formation in presphenoid Palatal shelves begin separation of oral and nasal cavity Meckel’s cartilage is rod-like across length of mandible Tooth development similar to mouse (tooth bud)
CS19	E64	E14.0	E16.0	Cranial base fully connected along the anterior–posterior aspect of head Lateral nasal capsule fuses with medial nasal septum. Palatal shelf fusion Dermal papillae and induction of the sensory vibrissae. VNO as an epithelial tube with thicker sensory epithelium in its ventromedial side Leaf-nose bud increases in size as lancet development begins
CS20	E70	E14.5	E16.5	Hypertrophic chondrocytes appear in the posterior basisphenoid Proliferation begins in the presphenoid
CS21	E75	E15.0	E17.0	Hypertrophic chondrocytes appear in anterior basisphenoid Ossification begins in the posterior basisphenoid
CS22	E80	E15.5	E17.5	Hypertrophic chondrocytes in anterior presphenoid Ossification in presphenoid Olfactory tract into olfactory epithelium
CS23	E85	E16.0	E18.0	Continued growth
CS24	E90	E16.5	E18.5/P1	Canine tooth appositional stage

We also obtained three embryos per stage across stages CS16–CS18 from the outgroup *M. natalensis* (courtesy of Dr. Nicola Illing, University of Cape Town, SA) to inform the direction of evolutionary changes among phyllostomid species comparisons. We collected embryos from the morphologically derived phyllostomid bats *A. jamaicensis* and *G. soricina,* with two biological replicates for CS17 and three biological replicates for CS18, each. We were unable to collect CS16 embryos for *A. jamaicensis* and *G. soricina* due to unexpected environmental conditions during our 2014–2017 field studies (see Additional file 3: Sampling). While not as comprehensively sampled as *C. perspicillata*, embryonic development in *M. natalensis, A. jamaicensis,* and *G. soricina,* as compared to that in mouse, all show a heterochronic shift in head versus limb development.

### Cellular proliferation

Proliferative cells were immunolabeled in all species with the molecular marker PH3 (phosphorylation of serine 10 residue at the N-terminal tail of histone H3). For each specimen, sagittal serial sections were obtained to capture the midfacial tissue between the eyes (Fig. 3). In all bat species, the expression of PH3 begins during the G2 phase of the cell cycle, peaks at metaphase and decreases at telophase (Additional file 2: Figure S2), similar to what has been previously characterized in other mammals [30, 43]. Generally, we observe an increase of PH3-signal when cells are in metaphase and a decrease of PH3-signal when cells are in telophase.

To evaluate proliferation during craniofacial development, the mean and median PH3-signal (cell-positive area) were scored for each embryonic stage of *C. perspicillata*. The base mean area of PH3 across all stages was 135.75 μm^2^ (cells sampled (*n*) = 86,584, Additional file 2: Figure S4) and similar ranges in cell size were observed at each stage (ANOVA, *p* < 2.2e−16, Additional file 2: Figure S4). To further examine the dynamics of cell size and developmental stage, the median size of PH3-signal during development was calculated as percent change from the previous stage (Additional file 2: Figure S3). Compared to the base mean area, cells are larger at stages CS20, CS23 and smaller at CS16–19, CS22, and CS24.

In *C. perspicillata*, the mean number of PH3-positive cells per stage from CS16 to CS24 is dynamic (Additional file 2: Figure S4) and the overall base mean in the number of PH3-positive cells is 1240 (cells sampled (*n*) = 86,584). Across development, compared to the base mean, proliferation is higher in stages CS16–17, CS18–19, and CS20–22 (Additional file 2: Figure S6). No change in proliferation is observed from CS17–CS18. However, cell proliferation, compared to the base mean, decreases substantially from stages CS19–20, CS22–23, and CS23–24 (ANOVA, *p* < 2.2e−16). As the *C. perspicillata* embryo progresses into the fetal stages (CS 22+), cell proliferation is further reduced.

### Growth zones during bat craniofacial development

**Fig. 4:**
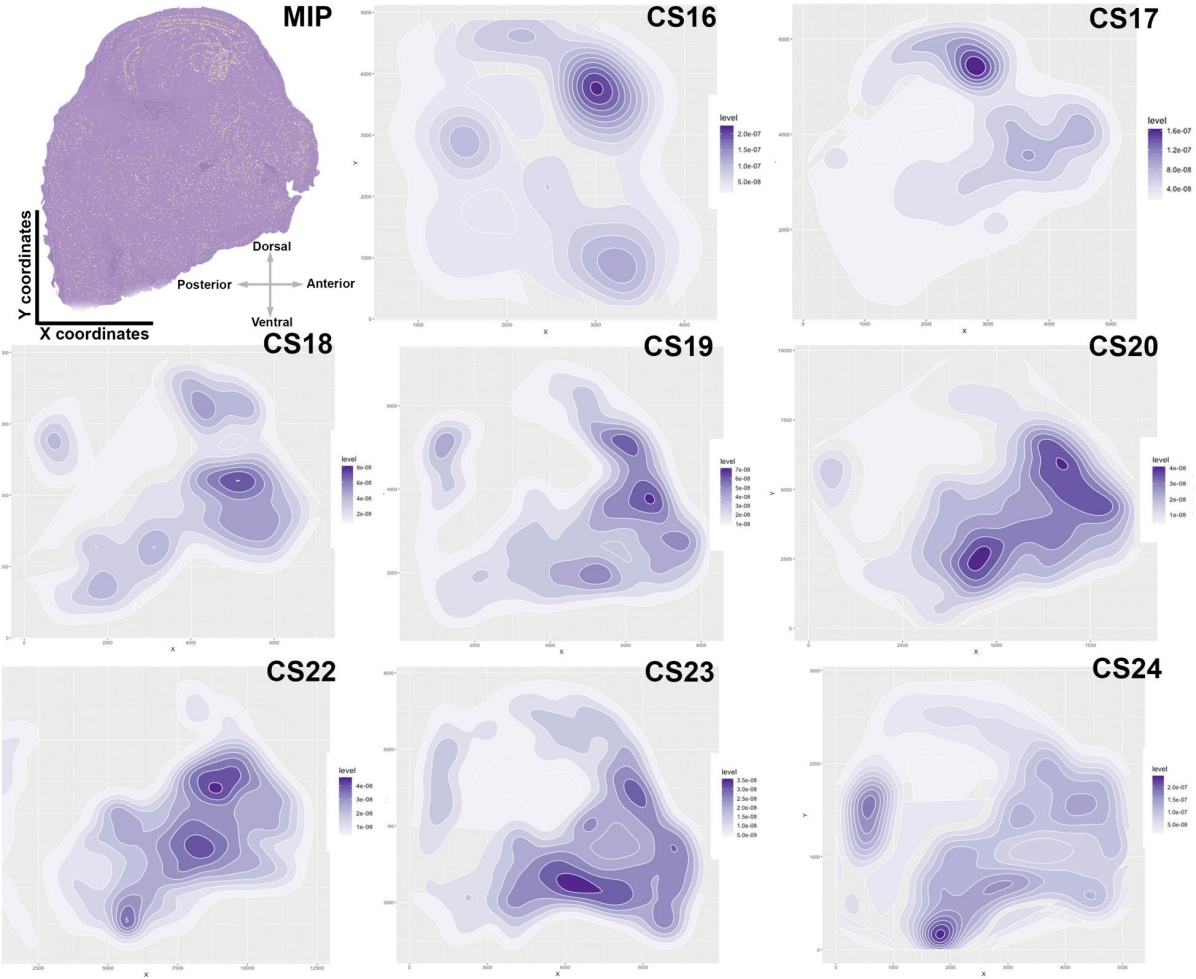
2D spatial distribution plots of PH3-positive cells during craniofacial development in *C. perspicillata*. A maximum intensity projection (MIP), obtained from an aligned stack of 2D sagittal sections for a CS17 embryo head, is shown in lateral view. All extracted *X*, *Y* coordinates from 2D image stacks are plotted as 2D distribution plots, oriented in lateral view. Thus, *X* axis relates to anterior–posterior position and the *Y* axis relates to dorsal–ventral position. All lateral images are oriented such that the anterior face points to the right and the back of the head points to the left. The intensity of purple color highlight concentrated regions of proliferation. At CS16, the majority of proliferation is localized to the forebrain. From CS17–CS24, growth zones appear in the midface, basicranium, hindbrain and forebrain

The 3D spatiotemporal distribution of proliferating cells along the anterior–posterior (A–P) axis can be summarized with 2D density distribution plots (Fig. 4). These plots quantitatively and qualitatively illustrate differences in the distribution of PH3-positive cells at discrete stages of craniofacial development. In *C. perspicillata*, a model bat, we collected a relatively large range of embryonic specimens (Additional file 1: Table S1) and our analysis of microscopic organization of the developing facial tissues demonstrates significant spatiotemporal differences in the pattern of cell proliferation (Fig. 4). At CS16, cell proliferation is localized to the ventricular zone of the ganglionic eminences in the developing brain. By CS17, the number of proliferating cells increases ninefold (Additional file 2: Figure S6), with intense proliferation in the brain ventricles, midface, and basicranium. At CS18, the overall number of proliferative cells decreases by 14% (Additional file 2: Figure S6), which is not statistically significant. At CS18, there is a noticeable decrease in forebrain cell proliferation and an increase in midfacial growth, adjacent to the developing forebrain. In addition, new areas of densely populated proliferative cells are observed in the hindbrain and along the basicranium. At CS19, as proliferating cells increase in number as the head grows, additional growth zones are found in the olfactory bulbs, lower jaw, tongue, and nose-tip. At CS20, the anterior growth of the upper midface correlates with increases in cell number dorsoventrally. By CS22, peak cell proliferation occurs, with the majority of divisions localized to facial tissues. The posterior growth of the midface, adjacent to the olfactory bulb, occurs in the developing cribriform plate and nasal septal cartilage. At CS23, cell proliferation decreases by 50%, with the majority of growth happening in the lower jaw and upper lip. At CS24, the number of proliferative cells drops by 64%, with the majority of growth remaining in the midface, hindbrain, and hyoid.

Qualitatively, the 2D plots of cell proliferation during craniofacial development in *C. perspicillata* reveal discrete cranial growth zones. To statistically assess these distribution patterns, we performed spatial distribution analysis with multi-distance K-means cluster analysis [36] along the anterior–posterior axis in *C. perspicillata*. We determined that proliferating cells are organized into at least four and as many as six optimal clusters (Additional file 2: Figure S7a). The six proliferation clusters represent distinct growth zones that match with the anatomical features qualitatively described for the 2D point patterns: forebrain, hindbrain, anterior midface, posterior midface, anterior basicranium and posterior basicranium (Figure S7b). The four detected major clusters are the forebrain, hindbrain, midface, and basicranium.

### Cellular dynamics within the growth zones

**Fig. 5:**
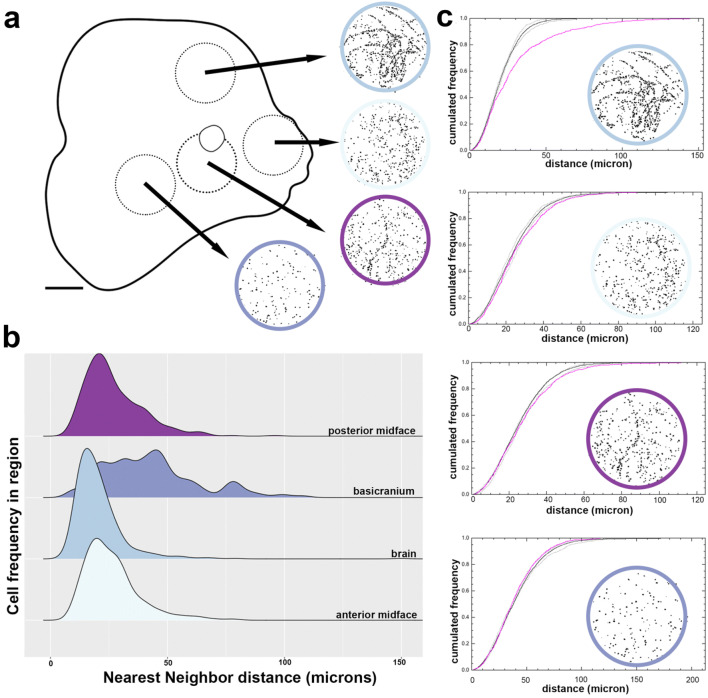
Growth zones and their spatial heterogeneity. Growth zones in the forebrain (blue), the anterior midface (light blue), the posterior midface (purple), and the basicranium (light purple) were investigated in our model bat *C. perspicillata*. A circular region with a radius of 550 μm across the four growth zones (**a**) were evaluated by nearest neighbor distance (NND) (**b**) and a Monte Carlo significance Goodness-to-fit test (F-function) (**c**). NND distribution between cells are shown as histograms. The mean NND is 26 μm in the anterior midface (*n* = 364), 27 μm in the posterior midface (*n* = 398), 20 μm in the forebrain (*n* = 570), and 42 μm in the basicranium (*n* = 147). Spatially, cells of the anterior midface, posterior midface and forebrain are distributed similarly. In the basicranium, cells are distributed homogenously. Pairwise comparisons between all regions using Wilcoxon rank sum test reveal the anterior and posterior midface have similar mean NND (*p* = 0.47), but the NND between other regions are unique (*p* < 2e−16) (**b**). For each growth zone, the distribution of PH3 cells were independently assessed (magenta lines in **c**). The estimated random F-function by Monte Carlo simulations (black line) and the 95% envelope (dotted black lines) describe random proliferation patterns. Deviations from randomness fall outside the 95% envelop and describe a structured proliferation pattern consistent with clustering (forebrain and midface). Deviations within the 95% envelop describe spatial randomness (basicranium) (**c**)

To further characterize the detected zones of elevated cell proliferation in *C. perspicillata*, we investigated cellular dynamics within a circular region (radius = 550 μm) spanning four such subareas, or growth zones, contributing to the size and shape of the midface: forebrain, anterior midface, posterior midface, and anterior basicranium (Fig. 5a). We examined compactness and spatial heterogeneity of cells by nearest neighbor distance (Fig. 5b) and a Monte Carlo significance goodness-to-fit test (F-function), respectively, as shown in Fig. 5c. In terms of compactness, variation is not normally distributed (Shapiro–Wilk *W* = 0.825, *p* < 0.000001). Therefore, we performed pairwise comparisons of compactness between regions using the Wilcoxon rank sum test, with adjusted p-values for multiple comparisons. This analysis shows that compactness of cells is similar within the anterior midface and posterior midface (*p* > 0.5) and within the brain and basicranium (*p* > 0.5). The compactness of cells is significantly different in the forebrain and midface (*p* < 2e−16) and the basicranium and midface (*p* < 2e−16). Within each growth zone, we assessed three different types of spatial heterogeneity: repulsion, attraction, and completely random distribution [4, 37, 46]. The anterior and posterior parts of midface and the forebrain show a spatial growth process consistent with attraction (closer together than expected, Fig. 5c). However, the basicranium proliferation is mostly contained within the 95% envelopes, consistent with random proliferation.

### Comparisons of growth zones among species

We find that *M. natalensis, A. jamaicensis,* and *G. soricina* have an overall qualitative spatial pattern of cell proliferation similar to *C. perspicillata* during craniofacial development (Figure S8) that falls into four regions: forebrain, midface, basicranium, and hindbrain (Additional file 2: Figure S7). However, there are several important peculiarities to the spatial distribution of cell proliferation among the species at CS18 (Additional file 2: Figure S8). In *M. natalensis*, three additional clusters of cell proliferation are present within the developing midface. In *G. soricina*, an additional growth zone is observed in the midbrain. In *A. jamaicensis,* PH3-positive cells also cluster in the olfactory bulb, adjacent to the forebrain. Additionally, while there is similar distribution in major areas of craniofacial development, the density of cells in each growth zone is locally modified (Additional file 2: Figure S8).

### Species-specific midfacial elongation is associated with differential cellular proliferation

To determine how and when the characteristic short faces of fruit bats and the long faces of nectar bats are generated during development, we examined embryos spanning a range of stages relevant to midfacial morphogenesis (Table 1). Due to the limited samples size of the wild-caught bat embryos, we incorporated museum-preserved embryonic specimens to supplement our morphometric dataset on facial length ratio (Additional file 2: Figure S9, Additional file 1: Table S2). The later batch also included embryos from additional phyllostomid species: *Macrotus waterhousii* with intermediate-length face (insect-feeding) and *Desmodus rotundus* with short-length face (blood-feeding), which we examined in our recent morphometric study [11]. These species allow for an even broader perspective on the species-specific cranial development among phyllostomid bats.

We report size-adjusted facial length measurements as the ratio between absolute facial length and total cranial length (Additional file 2: Figure S9, Additional file 1: Table S2). The phyllostomid bats *C. perspicillata, A. jamaicensis,* and *G. soricina* have more similar facial length (FL) to cranial length ratio (FL ratio = 0.28, 0.29, and 0.24, respectively) at CS17, whereas ecomorph-specific facial length differences emerge at CS18 in the fruit bats. In *C. perspicillata*, the FL ratio (0.26) remains relatively constant until birth (Additional file 2: Figure S9). In *A. jamaicensis*, facial length ratio (0.21) separates from other species until CS20, at which point facial length ratio reaches 0.18. In *G. soricina*, the facial length ratio remains relatively constant from CS18 until CS20, when it reaches a FL ratio of 0.21. We were unable to obtain CS21 stage embryos for these species. At CS22, the early stages of skeletal development, facial length ratio is similar again for *C. perspicillata* (FL ratio = 0.25), *A. jamaicensis* (FL ratio = 0.26), and *G. soricina* (FL ratio = 0.24). Unlike the constant facial growth observed in *C. perspicillata*, both *A. jamaicensis* and *G. soricina* undergo a burst of rapid growth to achieve the ~ 44% and ~ 11% increases in facial length at CS22, respectively. In *C. perspicillata* and *A. jamaicensis*, the facial length ratio is reduced to 0.21 at CS23. In *C. perspicillata*, facial length ratio increases and then stabilizes to ~ 0.24 at CS24 until birth. In *A. jamaicensis*, facial length ratio decreases to 0.16 at CS24 and the overall facial length ratio is maintained at ~ 0.16 until birth. In *G. soricina*, the facial length ratio is first reduced to 0.22 at CS24, after which point facial length ratio increases to 0.33 before birth.

**Fig. 6:**
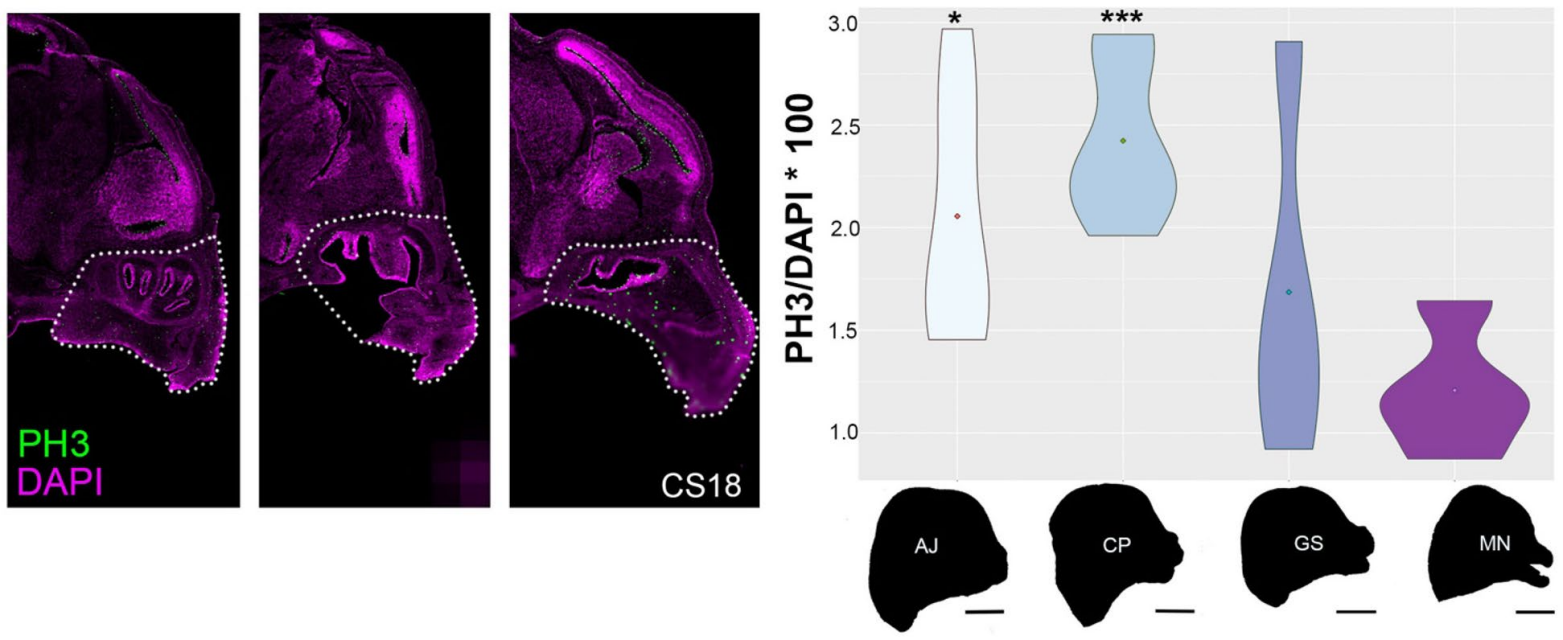
Pairwise comparisons of proliferation (PH3-positive cells ) between species. Standardized mean proliferation (PH3/DAPI * 100) were scored in the midface along the parasagittal plane at CS18. Shown on the left are three parasagittal serial sections for *A. jamaicensis*. The midface is outlined with a dotted line. Proliferation rates (right violin plots) were compared between *M. natalensis* (MN, 1.208, *n* = 3), *G. soricina* (GS, 1.686, *n* = 3), *C. perspicillata* (CP, 2.423, *n* = 3), and *A. jamaicensis* (AJ, 2.055, *n* = 3). ANOVA pairwise comparisons were performed with *M. natalensis* as the reference outgroup. Compared to *M. natalensis*, *G. soricina* has similar proliferation (*p* = 0.66), *C. perspicillata* has elevated proliferation (*p* = 0.001), and *A. jamaicensis* has slightly elevated proliferation (*p* = 0.027). All p-values are adjusted for multiple comparisons. Silhouettes of cranial morphology in lateral view are shown for each species at CS18, where species differences in facial length begin among phyllostomids. Scale bar: 2 mm

**Fig. 7:**
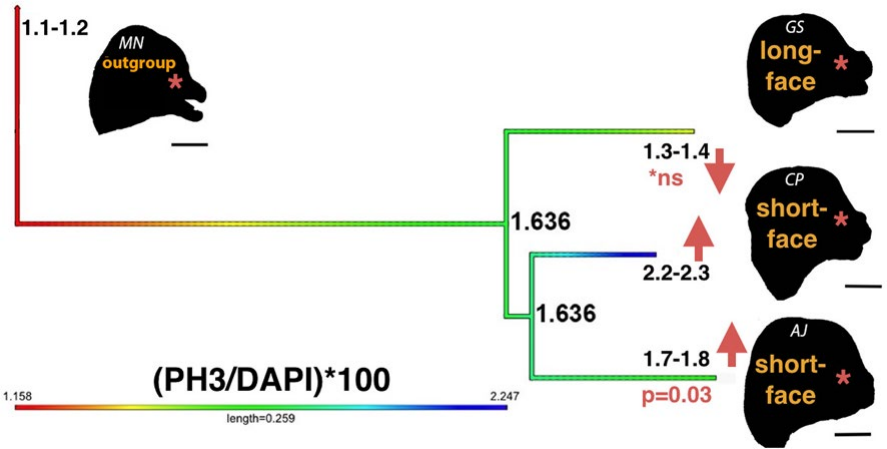
Ancestral state reconstruction of proliferation during development at CS18. Standardized proliferation (PH3/DAPI*100) was evaluated on a molecular tree pruned to relevant taxa (Fig. 2). Maximum likelihood ancestral state estimates at each node are shown for median proliferation rate and the evolutionary history is colored along each branch. Ancestral proliferation at each node was compared to modern species proliferation at the tips of the tree. The ancestral bat leading to *M. natalensis (MN)*, *G. soricina* (GS) and the ancestral bat leading to fruit-feeding bats have similar proliferation (1.636). In *M. natalensis*, proliferation is decreased (1.158). In the long-face of *G. soricina*, the estimated proliferation (1.332) is not statistically different (*p* > 0.05). Compared to ancestral proliferation, the growth process in the short-face of *C. perspicillata* (CP) is elevated to 2.247 (*p* = 0.001). In the short-face of *A. jamaicensis (AJ)*, compared to ancestral proliferation, the proliferation is slightly elevated to 1.755 (*p* = 0.03). Silhouettes of cranial morphology in lateral view are shown for each species and the proliferation data within the midface at CS18 likely relate to the growth zone (asterisk) shown in Fig. 7

We next compared the number of PH3-positive cells within the midface area in *M. natalensis*, *C. perspicillata*, *A. jamaicensis* and *G. soricina* at CS18 (Fig. 6). To control for differences in embryo sizes among these species, we performed a standardization on the mitotic cells by dividing the total number of PH3-positive cells by the total number of DAPI-positive cells and multiplied this by 100 (to obtain percent area fraction value). ANOVA pairwise comparisons between species are shown in Fig. 6, while a Tukey’s HSD (honestly significant difference) test identified differences between group means (Table 2). Cell proliferation in *C. perspicillata* (2.423, *n* = 3) is significantly elevated (*p* = 0.0006) compared to *M. natalensis* (1.208, *n* = 3). In *A. jamaicensis,* cell proliferation is significantly elevated (2.055, *n* = 3) compared to *M. natalensis* (*p* = 0.013), but not relative to other phyllostomid bats. Cell proliferation does not differ significantly between *G. soricina* (1.686, *n* = 3) and *M. natalensis* (*p* = 0.66, *n* = 3). To further evaluate cell proliferation differences between species, we analyzed them within a phylogenetic context. Since we had a small sample size and the range in proliferation rates within these samples was large (Additional file 2: Figure S4), we evaluated both the mean (Additional file 2: Figure S10) and median cell proliferation rates (Fig. 7).

**Table 2:** Tukey’s honest significance test

	Difference	Lower	Upper	*p* value adj
CP-AJ	0.368001827	− 0.265173035	1.00117669	0.384184404
GS-AJ	− 0.722892044	− 1.589902431	0.144118344	0.122976424
MN-AJ	− 0.847124335	− 1.634625627	− 0.059623043	0.032326069
GS-CP	− 1.090893871	− 1.928505482	− 0.25328226	0.008240205
MN-CP	− 1.215126162	− 1.970139066	− 0.460113259	0.001215846
MN-GS	− 0.124232291	− 1.083836944	0.835372361	0.982997469

### The cellular-level perspective on heterochrony

Further analysis of the cell proliferation patterns in the developing embryonic faces reveal that species- and ecomorph-specific differences in facial growth could be detected at the cellular level. The ancestral cell proliferation paradigm and the subsequent heterochronic changes in facial development were reconstructed from the embryonic cell proliferation data using square-change parsimony under a Brownian motion model of evolution (Additional file 2: Figure S8). Both mean and median trees from squared-change parsimony reveal that facial growth in *G. soricina* is similar to facial growth in *M. natalensis*, with both exhibiting parallel decreases in cell proliferation when compared to a common ancestor. In contrast, an increase in cell proliferation at the ancestral node leads to the short-faced fruit bats.

Finally, we estimated ancestral cell proliferation with maximum likelihood using the phytools *fastAnc* [63]. The mean (Additional file 2: Figure S10) and median (Fig. 7) values of cell proliferation represent two distinct evolutionary scenarios in the phyllostomid cranial history. First, the ancestral mean estimate of PH3/DAPI*100 at the nodes leading to *M. natalensis* and *G. soricina* is 1.755. From the ancestral condition, the cell proliferation in *M. natalensis* and *G. soricina* both decrease independently. The ancestral mean estimate of PH3/DAPI*100 at the node leading to fruit bats is 1.735. From this ancestral condition, the cell proliferation increases drastically in *C. perspicillata* and increases slightly in *A. jamaicensis*. Second, the median cell proliferation of PH3/DAPI*100 at the node for the most recent common ancestor of phyllostomids is estimated to be 1.636. Compared to the median ancestral cell proliferation, the proliferation of *G. soricina* does not differ significantly (1.3–1.4), whereas the proliferation rate in *C. perspicillata* is elevated from ~ 1.6 to 2.2–2.3 and the proliferation of *A. jamaicensis* is elevated from ~ 1.6 to 1.7–1.8.

## Discussion

Our recent morphometric studies on adult, juvenile and embryonic skulls revealed that heterochronic peramorphic shifts in craniofacial development contributed to much of the phyllostomid cranial diversity [11]. We hypothesized that differences in timing and/or rate of important cellular-level processes, such as cell proliferation, during embryonic and post-embryonic development in different phyllostomid lineages result in differential craniofacial growth and morphogenesis. This expectation was based on the wealth of developmental studies on craniofacial variation in laboratory mice reporting the central role of cellular proliferation dynamics in midfacial morphogenesis [34, 37, 46, 62] and on evolutionary developmental studies demonstrating cellular proliferation underlying species-specific facial differences [1, 2, 9, 33, 35, 75, 76]. The patterns of cellular proliferation we observed during craniofacial embryonic development align with our hypothesis and serve to better our understanding of the developmental mechanisms underlying the striking craniofacial diversity in phyllostomid bats.

The main dimension of phyllostomid cranial evolution is the length of the skull and snout, a morphological characteristic important to phyllostomid feeding ecology [5, 10, 15, 54]. The faces of phyllostomids show a full range of phenotypes from the extremely shortened-faces of the fruit bats to the highly elongated faces of the nectar-feeding bats, which encompass the majority of variation based on principal components (PC) analysis (Fig. 2). We chose three species both accessible in the wild and representative of the facial length variation captured along PC1: *Carollia perspicillata*, a predominantly piper-eating bat with an intermediate length cranium; *Artibeus jamaicensis*, a predominantly fig-eating bat with a short and wide face; and *Glossophaga soricina*, a primarily nectar-eating bat with an elongated head and narrow face. Related morphometric studies, each using different methodologies, identified embryonic stage CS18 as the starting point for morphological elaboration of phyllostomid skull shapes during development [11, 66]. In this study, we confirm that species- and ectomorph-specific facial length differences are first detected at stage CS18 for all the species studied (Additional file 2: Figure S9), with large differences in cell proliferation rates and distribution in the developing midface area at CS18 (Fig. 4, Additional file 2: Figure S8). Thus, we chose to focus on stage CS18 as the most informative time period at the onset of species differences in phyllostomid cranial shape for all further comparisons.

**Fig. 8:**
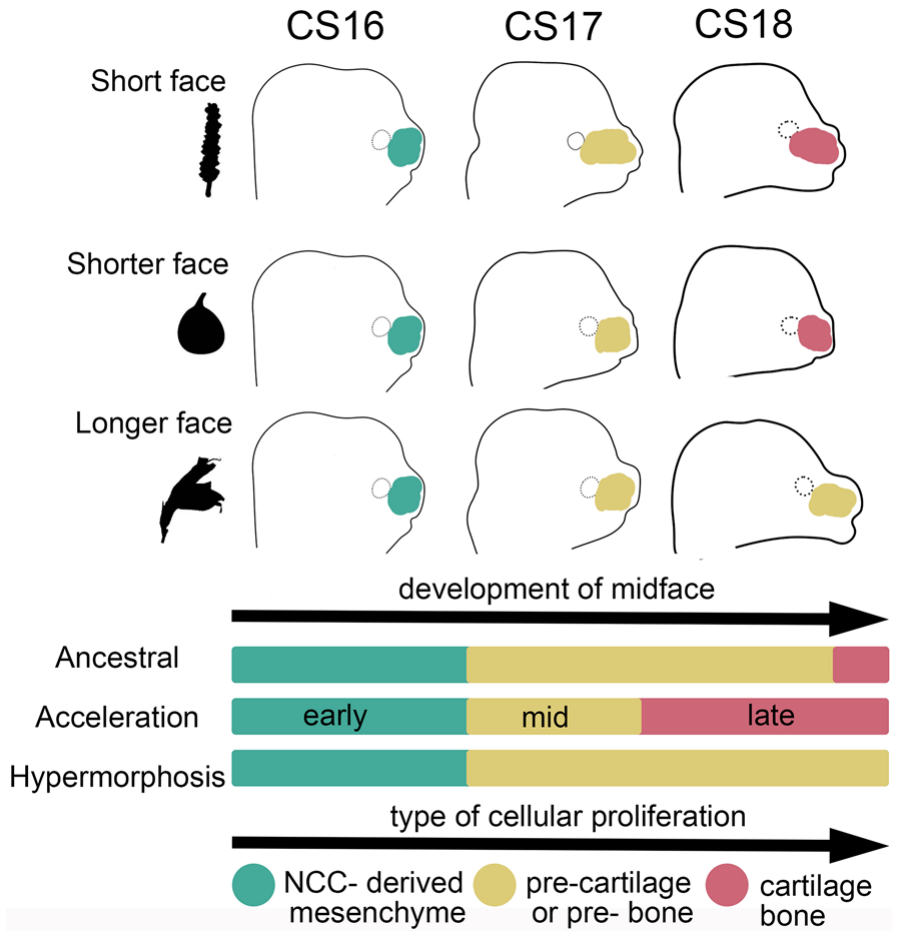
Proliferation model depicting extended development (peramorphosis) regulating facial length. We summarize key phases of craniofacial development between the short-faced *C. perspicillata*, the shorter face of *A. jamaicensis*, and the longer face of *G. soricina* at CS16 (early), CS17 (mid), and CS18 (late). The overall window of proliferation is the same duration of time between species. The colored regions of the midface represent cell proliferation and relate to aspects of facial development. In phyllostomid evolution, compared to the ancestor, skull shape is changed through heterochrony by increasing the rate of growth (acceleration) in fruit bats and by extending the duration of growth (hypermorphosis) in nectar bats. At the cellular level, the facial development is shown to undergo three phases of cellular division: early (green), mid (yellow), and late (red). Early divisions may relate to neural crest cells (NCC) during mesenchymal condensations (green) that give rise to pre-cartilage and pre-bone progenitors (yellow) and mature into cartilage and bone progenitors (yellow/red). Cartilage and bone cells proliferate and mature with a terminal division (red). With acceleration, elevated growth rate in committed progenitors (yellow) may trigger terminal divisions to occur (red) at an earlier time, which can then led to a shorter or truncated face. With hypermorphosis, constant growth rate (yellow) may relate to an extension in progenitor proliferation (delay in terminal division), which gives the face more time to grow and leads to a longer face. The figure is adapted from [71]

At stage CS18, relatively higher or lower level of cell proliferation is expected to play an important role in generating the correct size and shape of the developing midface. Ancestral state reconstruction of cell proliferation rates (Fig. 7) and distribution of proliferating cells within the midface region between species (*n* = 3 per species) provides a degree of explanation for the two models of peramorphosis suggested for short- and long-faced bats (Fig. 8). We find that the node leading to nectar bats and the node leading to fruit bats show similar cell proliferation (Fig. 7). This result is consistent with an idea from previous phylogenetic studies suggesting that all specialized feeding morphologies are derived from an ancestor with similar adult and developmental features [7, 16].

Interestingly, embryos of fruit bats have a higher rate of proliferation when compared to that of the calculated stage-matched ancestor (Fig. 7). The increased rate of development is expected to result in peramorphosis by acceleration [3]. Therefore, we propose that the increase in cell proliferation rate is the main proximal developmental mechanism associated with facial length development in fruit bats (Fig. 8). At the cellular level, elevated proliferation rate in committed progenitors may trigger terminal cell divisions to occur earlier, likely at CS18, which can then lead to a shorter or truncated face. This is in contrast to short-face morphology resulting from deficient outgrowth of the facial processes, the proximate mechanism proposed by Usui and Tokita [71]. We note that since *A. jamaicensis* is a basal branching member of the short-face fruit-eating bats, the more extreme short-faced fruit bats may indeed undergo additional heterochronic shifts as described by Usui and Tokita, 2018, but currently no data from morphometric studies support this potential paedomorphic change.

In contrast, the long-faced nectar bats demonstrate a proliferation rate similar to their calculated stage-matched ancestor (Fig. 7). Under the hypermorphosis model, the constant growth rate likely relates to a temporal extension in progenitor proliferation (i.e., delay in the offset of terminal cell division), which gives the face more time to grow—leading to a longer face (Fig. 8). After the stage CS18, their facial length remains relatively short until ~ CS22, when it begins to elongate (Additional file 2: Figure S9, Additional file 1: Table S2) and continues to elongate throughout fetal development. We propose that the development between stages CS18 and CS22 relates to a period of constant proliferation compared to other bat species. The relatively late onset of differential growth, observed as a change in proliferation rate and/or cellular differentiation, is likely associated with the observed peramorphosis by hypermorphosis in this phyllostomid ecomorph (Fig. 8). To further validate these hypotheses, more phyllostomid taxa and a higher number of embryos per species should be examined at the stages of development relevant to the differences in facial length (stages CS20, CS22, CS24).

In complex multi-part structures such as the vertebrate head, evolutionary changes, including those driven by heterochrony, can occur in a mosaic fashion as semi-independent modules [8, 21, 51]. One of the key aspects of heterochrony suggested by Gould [26] is evolutionary modularity [40]. In fact modularity, characterized by strong internal integration within each module and weak interactions with components of other modules, was demonstrated to be an important phenomenon in sequence heterochrony during mammalian skull evolution [24, 39, 57]. For this study, we consider stage CS18 as a good representative ontogenetic time point to study possible heterochronic modifications during craniofacial development. Our analysis of cell proliferation is primarily based on using *C. perspicillata* as a model, and we determined that the proliferating cells are organized into at least four and up to six optimal clusters (Additional file 2: Figure S7). These proliferative regions of the developing cranium belong to multiple tissues that develop along their own growth trajectories, in concert, to form the adult structures: the forebrain, hindbrain, posterior basicranium, anterior basicranium, posterior midface, and anterior midface.

When we assessed the growth trajectory pattern within the proliferative regions, we observed that the midface and forebrain both had an aggregated spatial distribution pattern, while the basicranium had a random spatial distribution pattern (Fig. 5). The aggregated pattern indicates a structured and directional model of cell proliferation. Indeed, both the forebrain and midface have been shown to develop in an inside-out organization; that is, growth at one basal surface displaces tissue to the apical surface through the movement of cells [53, 74]. For example, in the midface, the organized growth plate along the caudal end of the cartilage nasal septum “pushes” the septum anteriorly [74]. Similarly, the organized zones of progenitor cells found at the basal surface of the forebrain produce cells that migrate apically and expand the size of the cortex [47, 48, 53]. In contrast, the basicranium has dynamic and transient growth sites [46] that develop into an organized, bi-polar growth plate during the later stages of bone development [79]. Since we identify a random pattern of proliferation in the basicranium, we conclude the organized growth sites have not yet appeared by stage CS18.

While we directly measured cell proliferation differences within a phylogenetically meaningful comparative framework, we cannot yet rule out other potential cellular mechanisms contributing to differential cranial growth, such as changes in cell size, shape and movement. Additionally, we chose to focus our comparative analysis at embryonic stage CS18, and we do not evaluate in detail the entire range of possible cellular heterochronies. However, we do expect to find local or mosaic cellular-level heterochronies during craniofacial morphogenesis, as suggested by the detailed 2D cell proliferation maps for *C. perspicillata.*

This descriptive study finds correlations between differential growth of macroscopic structures and the behavior of cells at the microscopic level that both contribute to morphogenesis. Our cellular proliferation data in the developing midface are largely interpreted within the framework and with *a priori * knowledge of peramorphosis, a mode of heterochrony previously suggested to play an important role in the evolution of phyllostomid facial diversity. Due to the relatively small sample sizes and limited taxonomic sampling available for this study, it is too early to conclude that the reported heterochronic modifications to cell proliferation patterns can explain craniofacial evolution of the phyllostomid radiation in its entirety. However, our previous morphological studies strongly suggest that the species we selected for this study are indeed representative of the Glossophaginae nectar bats, the Carollinae fruit bats,  and for the Stenodermatinae short-faced fruit bats. Additional taxa, particularly those representing the basal insect-feeding phyllostomids, vampire bats, and the more morphologically extreme nectar and fruit-eating bats, will need to be investigated to understand more comprehensively how differential cell proliferation contributes to cranial morphogenesis. Likewise, extending these comparative analyses to the molecular and genetic levels should reveal precise developmental mechanisms involved in the evolution of phyllostomids.

## Conclusion

Comparing cell proliferation rates and patterns to an observed phenotypic variation is a valuable approach for investigating heterochronic shifts in growth and resulting morphological evolution. Quantitative maps of proliferation during development show how craniofacial growth occurs at stereotypical locations in the craniofacial region. Our fine-scale morphogenetic analysis of bat embryos with distinct faces emphasizes how facial length changes correlate with altered cellular proliferation to the midface. Ancestral state reconstructions of cell proliferation in the most recent common ancestor of Noctilionoidea suggest that the elevated cell proliferation in fruit bats is an apomorphic trait and the lower cell proliferation in nectar bats is plesiomorphic. Variation in the rate and/or duration of cell proliferation during craniofacial development appears to be an important proximal mechanism of heterochronic growth that facilitated evolutionary diversification of phyllostomid faces. Furthermore, the widespread employment of heterochrony implicated by many evolutionary developmental studies on mammals and other vertebrate animals [14, 21, 23, 42, 51, 52, 69] implies that similar heterochronic changes at the cellular level are broadly important in morphological evolution.

## Experimental procedures

### Field work

*Miniopterus natalensis* (South Africa), *Carollia perspicillata* (Trinidad), *Artibeus jamaicensis* (Trinidad), and *Glossophaga soricina* (Trinidad) were wild collected under permits issued by the Western Cape Nape Nature Conservation Board (South Africa) and the Wildlife Section, Forestry Division, Ministry of Agriculture, Land and Marine Resources (Republic of Trinidad and Tobago). *M. natalensis* pregnant females were originally collected in September to October of 2008 from the De Hoop Guano Cave in South Africa for studies in bat limb development [32, 55]. Cranial tissues unused in bat limb studies were generously shared by Professor Nicola Illing (University of Cape Town). *C. perspicillata*, *A. jamaicensis*, and *G. soricina* pregnant females were collected from reproductively synchronized, wild populations living on Trinidad (Additional file 2: Figure S11) during the March to May for studies of embryonic development. In the case of *C. perspicillata* (the best studied of the three species), this period would overlap portions of two successive, synchronized reproductive periods exhibited by most adult females in the population [58, 59]. Pregnant females were humanely caged and transported, and generally euthanized within 6 and 10 h after collection.

### Embryo collection and storage

Immediately after euthanasia of the mothers, their embryos (one per female) were harvested as described previously [60]. Embryos were gently rinsed in chilled 1× phosphate buffered saline (PBS), pH 7.4 (Invitrogen), and quickly staged based on limb development features according to a Carnegie staging table [13, 32, 67]. Embryos were perfused intracardially with chilled 1× PBS followed by perfusion with chilled 4% paraformaldehyde in 1× PBS (PFA). Tissues were then incubated in 4% PFA overnight at 4 °C. Post-fixation, tissues were rinsed three times in chilled 1× PBS for 5 min, then dehydrated through a methanol series (25%, 50%, 75% in 1× PBS and 100%) for 20 min each wash, and stored in 100% methanol at − 20 °C [61]. Embryonic heads were stored separately from bodies. For *Carollia perspicillata*, we were able to obtain tissues spanning stages CS16–CS24. For *Miniopterus natalensis*, we were able to obtain tissue spanning stages CS16–CS18. We obtained tissue at CS17–CS24 for *A. jamaicensis* and *G. soricina*, but were not able to obtain many biological replicates. Therefore, stages with less than two specimens were excluded from analysis. Tissues were exported with the permission of the Wildlife Section, Forestry Division of the Ministry of Agriculture, Land and Fisheries of the Republic of Trinidad and Tobago and imported with the permission of U.S. Fish and Wildlife Service.

### Immunohistochemistry

Bat embryonic cranial tissue were rehydrated to 0.1 M PBS, cryo-protected in 10% sucrose overnight at 4 °C, followed by 30% sucrose wash overnight at 4 °C, and then another overnight wash in 1:1 sucrose: OCT mix (Tissue-Tek; Sakura Finetek USA) at 4 °C. Tissue was briefly embedded in OCT for 30 min at 4 °C prior to flash freezing on dry ice. To maximize molecular and morphological information, we serially sectioned embryo heads at 10 μm with a cryostat (Leica CM3050S) along the sagittal plane to capture craniofacial length. Serial 10 μm sections were collected every 100 μm and mounted onto glass suprafrost slides (Fisher; Fig. 3). One series was stained for histological reference for archiving into the Museum of Comparative Zoology at Harvard. Another series was used to assay proliferation. All remaining serial sections were stored in − 80 °C freezer for future experiments. For antigen unmasking, the slides were steamed in pre-warmed 1 mM sodium citrate buffer with 0.05% Tween20, pH 6.0 for 30 min. Sections were blocked in 10% donkey serum (Gibco), 0.1% Tween20, and 0.3 M glycine. Immunodetection was performed with a primary antibody targeting PH3 (1:1000, Millipore 06-570) and incubated for 24 h at 4 °C. Sections were rinsed in 1× PBS (phosphate buffered saline) and incubated for 1 h with a secondary antibody conjugated with Cy2 (1:200, Jackson Lab). Antibodies were diluted in 1% donkey serum with 0.01% Tween20 and 0.03 M glycine. The sections were counterstained with DAPI (4’,6-diamidino-2-phenylindole) fluorescent DNA stain (1:1000, ThermoFisher D1306).

### Fluorescent imaging

All embryo heads are imaged with the snout pointing to the right. Serial sections of the entire head were captured with a wide-field scanning microscope (Olympus VS120) at 20× magnification. Imaging large fields of view was achieved by imaging multiple, smaller images and combining them to a larger overview, which were automatically aligned by the imaging software (Olympus). All imaging was done at the Harvard Medical School Neurobiology Imaging Facility at the Department of Neurobiology.

### 3D image visualization

Sagittal sections of immunostained tissue representing medial–lateral aspects of the head (Fig. 3) were imaged, aligned, and reconstructed as a virtual image stack in Fiji [65]. Original image formats from Olympus VS120 were imported into FIJI using Bio-formats importer and saved as a 16-bit TIF. Each 10 μm serial section spanning the medial–lateral aspects of the head were combined as 2D stack of images with each 10 μm serial section restricting data to a particular spatial plane of focus along the medial–lateral aspects of the head. The distance between each serial section of a series is 100 μm, so the depth data is set to 100 μm. Since the 2D images have depth data, the 2D stack contains data as *X*, *Y*, *Z*. To reconstruct an 3D object, the 2D images were aligned by two Procrustes transformations, translation and rotation, with the FIJI Plugin *StackReg*.

### Cell segmentation

Aligned 2D stacks of unprocessed 16-bit fluorescent image data were used for quantitative analysis. Each channel in the aligned 2D stack was similarly processed. Each image channel of the 2D stack was isolated and analyzed separately as outlined in (Additional file 2: Figure S12) with a custom java script. Individual cells were segmented (i.e., detected) with the watershed-based function with a noise tolerance of 100 within the FIJI module *Find Maxima*. Since PH3 is a nuclear marker, cell signal was relatively compact in the center of each cell, which is ideal for the *Find Maxima* function. Each maxima generates a binary segmented particle with *X*, *Y* coordinates. To define the area occupied by cell signal, a threshold was adjusted on the original 16-bit image with an Otsu algorithm to minimize intraclass variance of the black (background) and white (signal) intensity. Thresholding generates a binary image defining the total area of cell signal. Individual cell signal was generated by combining the segment binary image with the threshold binary image.

### Object-based quantification

Object-based identification of PH3-positive cells used for analysis is described in the supplemental text and Additional file 2: Figure S12. Cell information (position, size, shape) was quantified using a custom script in FIJI. Positional information coordinates (*X*, *Y*) of each cell center maxima during mitosis was obtained. Size of each cell is presented as the area. Shape descriptors are used to help evaluate cellular behavior (i.e., a round cell likely G2 or prophase and an elongated cell likely metaphase or telophase). Roundness of PH3-signal was determined by dividing the major axis of the cell body with the minor axis of the cell body  (Figure S13).

### Spatial pattern analysis

The extracted positional information (*X*, *Y*) of detected objects in a 2D image stack was used to generate a point pattern in R. The 3D spatial distribution of proliferating cells is represented as a 2D maximum intensity projection of density data on 2D contour plots for easy visualization, where dark regions relate to concentrated cells and lighter regions are less dense.

Multi-distance K-mean cluster analysis assessed clustering patterns among the entire distribution of coordinates. K-means cluster analysis finds groups in data without defined categories, with the number of groups defined by the variable K. The unsupervised learning works iteratively to assign data points to a group based on feature similarity. The collection of features which define a group are used to interpret what type of group each cluster represents. We evaluate a range of *K* values with the commonly used visual metric, the “elbow point”. Increasing the K value will always decrease the mean distance between points in a cluster, so the ideal K value is where the rate of decrease sharply shifts, or bends.

A subset of concentrated regions of growth identified by 2D contour plots and *K*-means cluster analysis were further analyzed by nearest neighbor distance analysis and a Monte Carlo significance goodness-to-fit test (F-function). These four regions are shown in Fig. 5. The spatial statistics were evaluated within a circular region with a radius of 550 μm spanning subareas, or growth zones: forebrain, anterior midface, posterior midface and anterior basicranium. Nearest neighbor analysis measures the closest distance between each point and then compares it to a random sample of points to aide in evaluating the distribution pattern of cells. Cells closer together than expected from random are clustered. Additional patterns of distribution are random or dispersed.

The F-function evaluates distribution pattern deviation from spatial randomness to determine the statistical significance of clustering patterns. For each cluster (i.e., growth zone), random shuffling of point patterns within the reference region is used to generate a random distribution pattern. This randomization is done five times to generate an average random distribution. A second set of random point patterns is then generated to calculate the expected variation around the average random distribution. The distribution pattern for each cluster is plotted alongside the average random distribution. The distribution pattern can fall within the expected variation around the average random distribution, which is then interpreted as a random distribution pattern. The distribution pattern can shift ahead of the random distribution, which is then interpreted as attraction (closer together than expected). The distribution pattern can shift behind the random distribution, which is then interpreted as repulsion (further apart than expected).

### Ancestral state reconstruction

The proliferation data between species was normalized to account for size differences. To standardize, the total amount of PH3-positive cells was divided by the total amount of DAPI-positive cells and multiplied by 100 (percent area fraction). Group mean and median proliferation were mapped onto the tips of the phylogeny shown in Fig. 2. The phylogeny used was a pruned tree from [11], which was based on the molecular phylogeny from [15]. Estimates of ancestral states were performed with parsimony using Mesquite [49] and performed with maximum likelihood using *Phytools fastAnc* [63] in R. The ancestral states were visualized as a color gradient representing continuous values projected onto the phylogeny.

## Supplementary information

**Additional file 1: Additional Tables.** (1) Specimens collected, (2) Facial Length ratio.

**Additional file 2: Additional Figs.** (1) Heads matched to same stage in mouse & bat, (2) PH3 cell panel, (3) Delta change of cell size, (4) Base mean summary area CP PH3, (5) MN PH3 cell size mean, (6) Percent change number of cells (count), (7) K-mean cluster, (8) CS18 ASR distribution map, (9) Facial length ratio, (10) ASR Mean Proliferation, (11) Sampling sites, (12) Cell segmentation, (13) PH3 CP & MN cell size vs shape.

**Additional file 3.** Supplemental methods: (1)Sampling, (2) Maximizing experiments with few biological replicates, (3) Automatic cell quantification (4) PH3 as a mitotic marker, (5) Cellular development.

## Data Availability

Data analyzed during this study are included in this article and in the supplementary information. Reference specimens are deposited as a Trichrome series into the Museum of Comparative Zoology (MCZ) at Harvard. All raw and processed 2D stack-images of imaged Trichrome (40×) and immunohistochemistry (20x) are available to others for analysis. We have focused on the midface, but we hope others will be interested in analyzing other aspects of craniofacial development. In addition, body and limbs for all specimens (Additional file 1: Table S1) are stored in methanol at − 20 °C and are available upon request and might eventually be acquisitioned into the MCZ for archiving.
