# Supplementary material for: Differential cellular proliferation underlies heterochronic generation of cranial diversity in phyllostomid bats
Source: EvoDevo. 2020 Jun 2;11:11. doi: 10.1186/s13227-020-00156-9 (PMC7268441; doi:10.1186/s13227-020-00156-9)
Supplement: Supplementary file 1 — Additional file 1: Additional Tables. (1) Specimens collected, (2) Facial Length ratio. [file 13227_2020_156_MOESM1_ESM.docx]

# Additional Table

1. Specimens collected (50 embryos)

| Stage | Species | Specimen ID |
| --- | --- | --- |
| CS16 | *Carollia perspicillata* | 2005-101 |
| CS16 | *Carollia perspicillata* | 2005-168 |
| CS16 | *Carollia perspicillata* | 2007-126 |
| CS16 | *Miniopterus natalensis* | 2008-085 |
| CS16 | *Miniopterus natalensis* | 2008-080 |
| CS16 | *Miniopterus natalensis* | 2008-075 |
| CS17 | *Carollia perspicillata* | 2005-056 |
| CS17 | *Carollia perspicillata* | 2006-176 |
| CS17 | *Carollia perspicillata* | 2016-222 |
| CS17 | *Miniopterus natalensis* | 2008-066 |
| CS17 | *Miniopterus natalensis* | 2008-048 |
| CS17 | *Miniopterus natalensis* | 2010-009 |
| CS17 | *Artibeus jamaicensis* | 2017-317 |
| CS17 | *Artibeus jamaicensis* | 2017-346 |
| CS17 | *Glossophaga soricina* | 2016-249 |
| CS17 | *Glossophaga soricina* | 2017-343 |
| CS18 | *Carollia perspicillata* | 2005-110 |
| CS18 | *Carollia perspicillata* | 2005-071 |
| CS18 | *Carollia perspicillata* | 2009-199 |
| CS18 | *Miniopterus natalensis* | 2008-074 |
| CS18 | *Miniopterus natalensis* | 2008-076 |
| CS18 | *Miniopterus natalensis* | 2008-081 |
| CS18 | *Artibeus jamaicensis* | 2017-311 |
| CS18 | *Artibeus jamaicensis* | 2017-324 |
| CS18 | *Artibeus jamaicensis* | 2018-403 |
| CS18 | *Glossophaga soricina* | 2016-250 |
| CS18 | *Glossophaga soricina* | 2015-140 |
| CS18 | *Glossophaga soricina* | 2016-245 |
| CS19 | *Carollia perspicillata* | 2004-135 |
| CS19 | *Carollia perspicillata* | 2011-141 |
| CS19 | *Glossophaga soricina* | 2014-019 |
| CS19 | *Artibeus jamaicensis* | 2016-253 |
| CS20 | *Carollia perspicillata* | 2016-257 |
| CS20 | *Carollia perspicillata* | 2005-021 |
| CS20 | *Carollia perspicillata* | 2005-011 |
| CS20 | *Artibeus jamaicensis* | 2017-319 |
| CS20 | *Glossophaga soricina* | 2017-308 |
| CS22 | *Carollia perspicillata* | 2015-102 |
| CS22L | *Carollia perspicillata* | 2012-127 |
| CS22 | *Artibeus jamaicensis* | 2013-004 |
| CS22 | *Glossophaga soricina* | 2016-268 |
| CS22L | *Glossophaga soricina* | 2013-003 |
| CS23 | *Carollia perspicillata* | 2016-240 |
| CS23 | *Carollia perspicillata* | 2013-126 |
| CS23 | *Carollia perspicillata* | 2013-122 |
| CS23 | *Artibeus jamaicensis* | 2017-345 |
| CS24 | *Carollia perspicillata* | 2015-103 |
| CS24 | *Carollia perspicillata* | 2016-230 |
| CS24 | *Glossophaga soricina* | 2016-251 |
| CS24 | *Artibeus jamaicensis* | 2013-006 |

1. Facial length (FL) to cranial length (CL) ratio from CS16 until adulthood.

| Stage | Source | Species | FL:CL average |
| --- | --- | --- | --- |
| CS16 | Wild caught | CP | 0.1925195 |
| CS16 | Wild caught | MN | 0.2853486 |
| CS17 | Wild caught | AJ | 0.28912068 |
| CS17 | Wild caught | CP | 0.27957149 |
| CS17 | Wild caught | GS | 0.24259472 |
| CS17 | Wild caught | MN | 0.32196061 |
| CS18 | Museum | AJ | 0.17892669 |
| CS18 | Wild caught | AJ | 0.21506985 |
| CS18 | Museum | CP | 0.14412605 |
| CS18 | Wild caught | CP | 0.25492051 |
| CS18 | Wild caught | GS | 0.24100119 |
| CS18 | Wild caught | MN | 0.35066744 |
| CS19 | Museum | AJ | 0.15482535 |
| CS19 | Wild caught | CP | 0.24872165 |
| CS19 | Museum | GS | 0.23035969 |
| CS19 | Wild caught | GS | 0.22515965 |
| CS19 | Museum | MW | 0.16739794 |
| CS20 | Wild caught | AJ | 0.18191378 |
| CS20 | Wild caught | CP | 0.25935585 |
| CS20 | Museum | GS | 0.24927463 |
| CS20 | Wild caught | GS | 0.20529328 |
| CS21 | Museum | CP | 0.16571934 |
| CS22 | Museum | AJ | 0.14857496 |
| CS22 | Wild caught | AJ | 0.2627907 |
| CS22 | Wild caught | CP | 0.24623956 |
| CS22 | Wild caught | GS | 0.24163571 |
| CS23 | Museum | AJ | 0.14209756 |
| CS23 | Wild caught | AJ | 0.21636356 |
| CS23 | Museum | CP | 0.17412736 |
| CS23 | Wild caught | CP | 0.2177067 |
| CS23 | Museum | GS | 0.26985646 |
| CS23 | Museum | MW | 0.22155126 |
| CS24 | Museum | AJ | 0.15271829 |
| CS24 | Wild caught | AJ | 0.1573948 |
| CS24 | Museum | CP | 0.16833283 |
| CS24 | Wild caught | CP | 0.23729848 |
| CS24 | Museum | DR | 0.18558982 |
| CS24 | Museum | GS | 0.26690906 |
| CS24 | Wild caught | GS | 0.21936296 |
| CS25* | Wild caught | CP | 0.24352953 |
| CS25* | Wild caught | GS | 0.27075227 |
| CS25* | Museum | CP | 0.1734128 |
| CS26* | Wild caught | CP | 0.23021554 |
| CS26* | Museum | DR | 0.17756544 |
| CS26* | Wild caught | GS | 0.33244478 |
| CS26* | Museum | AJ | 0.16810012 |
| CS26* | Museum | DR | 0.15887664 |
| CS28* | Museum | AJ | 0.17798881 |
| CS28* | Museum | CP | 0.19507408 |
| CS28* | Museum | DR | 0.18749926 |
| CS30* | Museum | AJ | 0.20449587 |
| CS30* | Museum | CP | 0.19846922 |
| CS30* | Museum | DR | 0.15781478 |
| CS30* | Wild caught | DR | 0.22970354 |
| CS30* | Museum | GS | 0.31561249 |
| CS30* | Museum | MW | 0.21776412 |
| ADULT | Museum | AJ | 0.20949974 |
| ADULT | Museum | CP | 0.25959676 |
| ADULT | Museum | DR | 0.12783347 |
| ADULT | Museum | MW | 0.26055757 |
| ADULT | Museum | GS | 0.37958642 |
| ADULT | Museum | MN | 0.39954371 |
