# Supplementary material for: Differential cellular proliferation underlies heterochronic generation of cranial diversity in phyllostomid bats
Source: EvoDevo. 2020 Jun 2;11:11. doi: 10.1186/s13227-020-00156-9 (PMC7268441; doi:10.1186/s13227-020-00156-9)
Supplement: Supplementary file 2 — Additional file 2: Additional Figs. (1) Heads matched to same stage in mouse & bat, (2) PH3 cell panel, (3) Delta change of cell size, (4) Base mean summary area CP PH3, (5) MN PH3 cell size mean, (6) Percent change number of cells (count), (7) K-mean cluster, (8) CS18 ASR distribution map, (9) Facial length ratio, (10) ASR Mean Proliferation, (11) Sampling sites, (12) Cell segmentation, (13) PH3 CP & MN cell size vs shape. [file 13227_2020_156_MOESM2_ESM.docx]

# Additional Figures


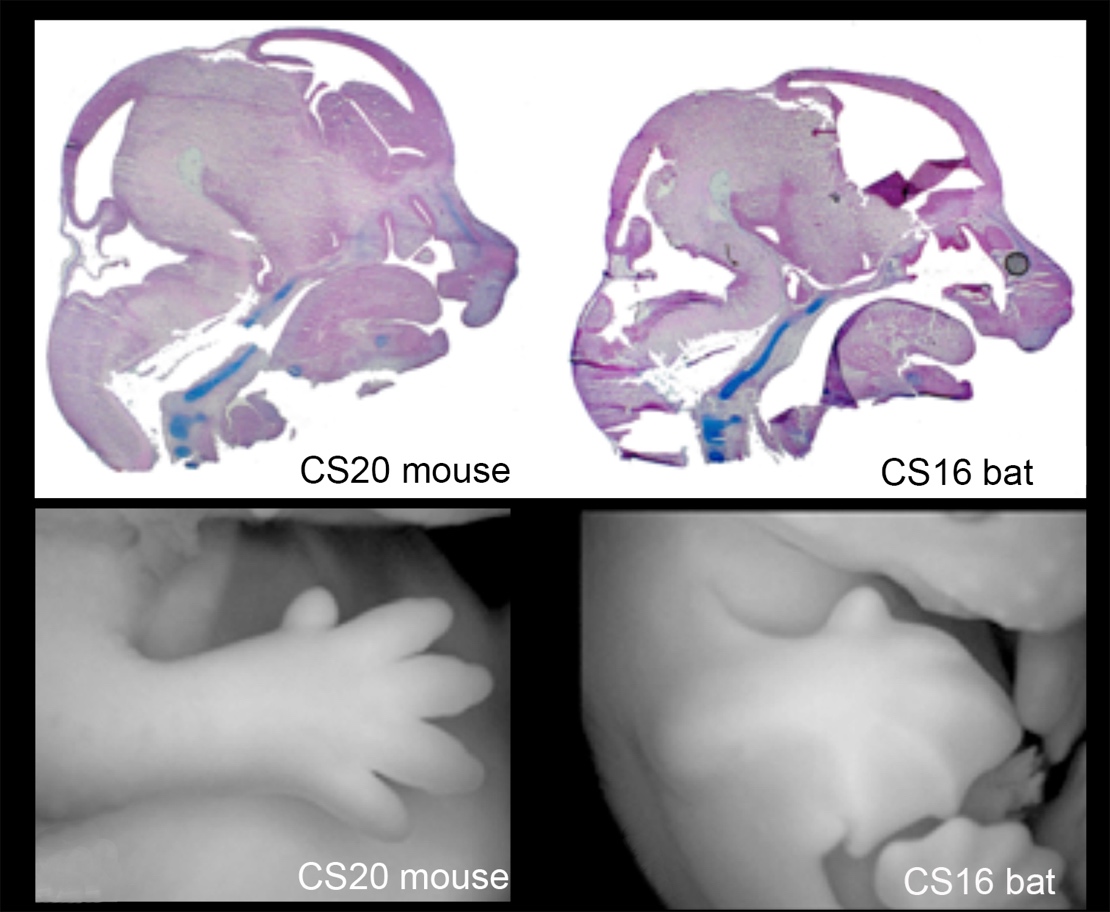

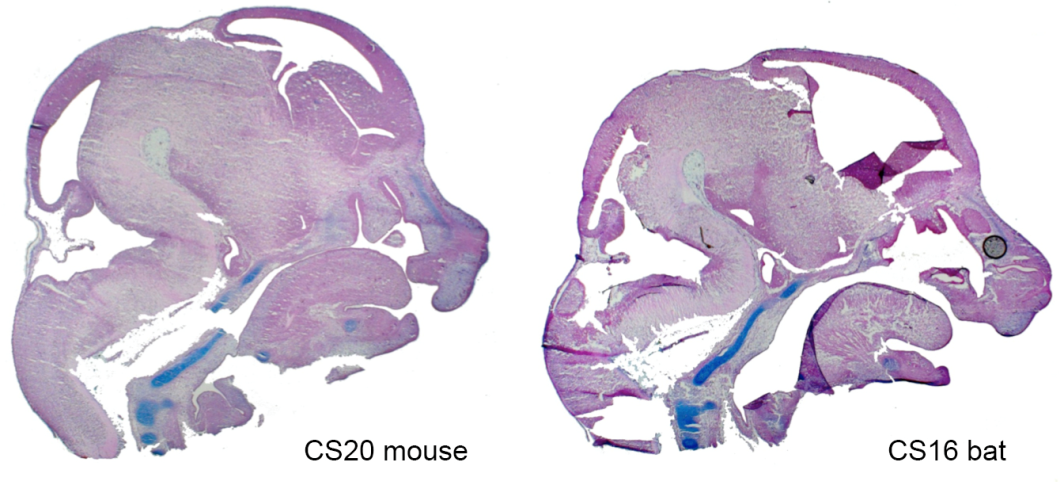


## **Figure S1:**

**Head and limb development differ**. H&E histology with alcian blue counterstain of a CS20 mouse embryo (~14.5dpc) matches that of a CS16 *C. perspicillata* embryo at the equivalent parasagittal plane. Cranial base development was used to match head development. Carnegie stage was assigned based on the progression of limb development, so while the CS16 limbs of bat match the CS16 limbs of mouse (Behringer et al. 2009), the mouse head is less developed or bats accelerate their craniofacial development. Whole-mount images of limbs from Figure 2 of Behringer et al. 2009.


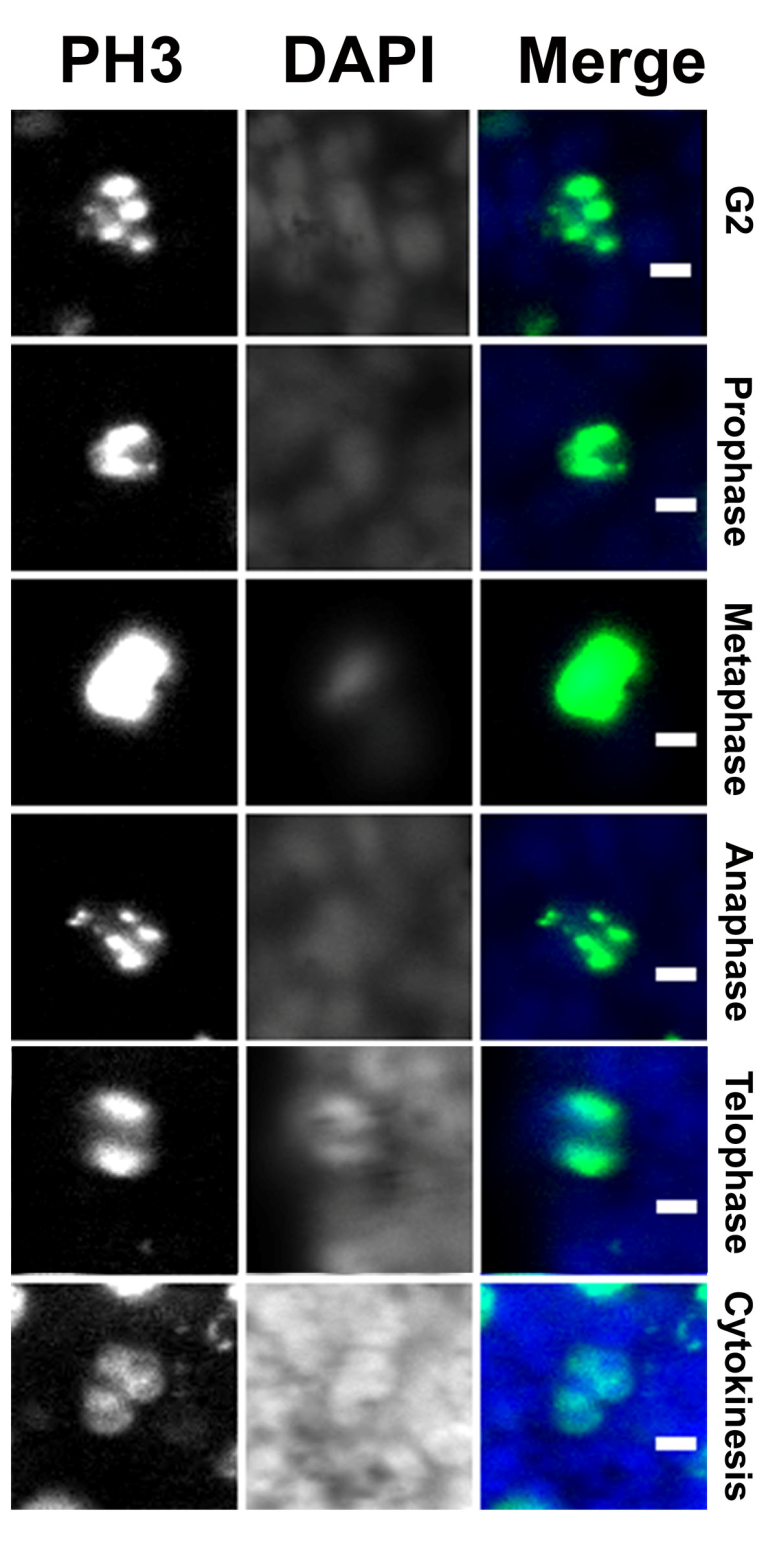


## **Figure S2:**

**PH3 as a mitotic marker.** Localization of phosphohistone H3 (PH3, green) during mitosis in bat cells. The size (area) of PH3-signal per cell is related to the phase of the cell cycle. DNA is stained with DAPI and aides in visualizing condensed chromatin during mitosis to identify the cell-cycle progression (G2, prophase, metaphase, anaphase, telophase, cytokinesis). The PH3-signal peaks at metaphase and is weakest during cytokinesis. Scale bar: 5µm.


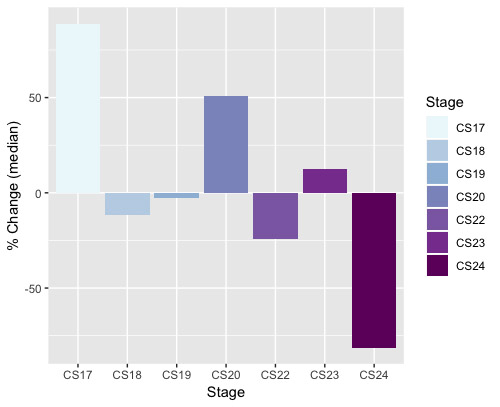


## **Figure S3:**

**Percent change in the median size (area) of PH3-signal per cell from CS16 to CS24.** The amount of PH3-signal per cell was quantified at each stage of development in *C. perspicillata*. The percent change in proliferation at each stage is calculated from the preceding stage. There is an 88% increase in median cell area labeled with PH3, at CS17 (125.87µm, n=5674), which then slightly decreases to 111.07µm at CS18 (n=4878) and CS19 (n=16765). A 50% increase in the median area is observed at CS20. At CS22, the size of PH3 signal decreases by 24% to 123.45µm (n=30274) and by 12% at CS23 (n=15651). By stage CS24, PH3 signal per cell decreases by 81% to 26µm. The peak median size (area) occurs at CS20 (162.89µm, n=7155) and the smallest median size (area) occurs at CS24 (26µm, n= 1033).


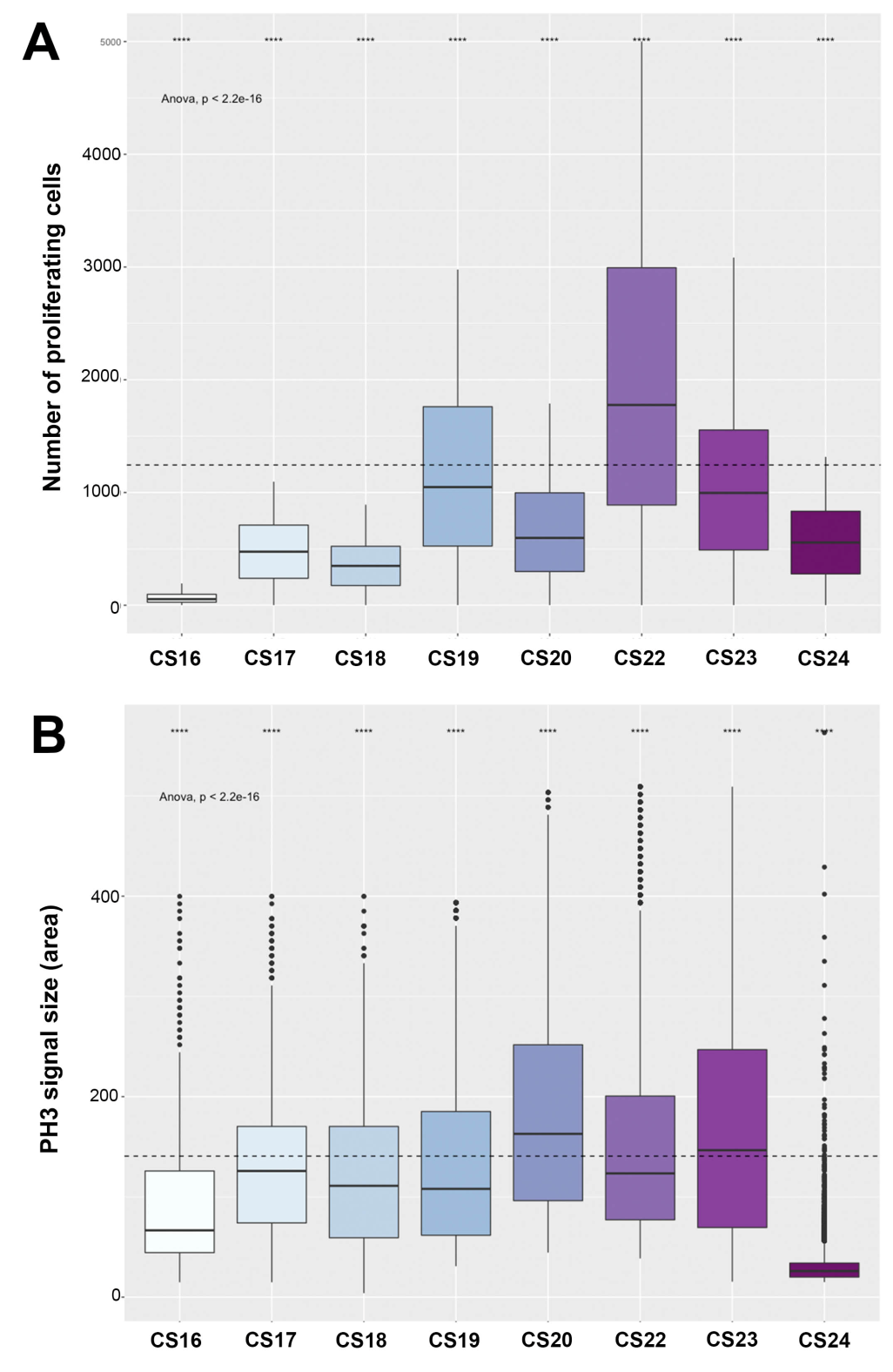


## **Figure S4:**

**Proliferation during CS16-CS24 craniofacial development in the model bat *C. perspicillata*.** The number of dividing cells and area of signal per cell was quantified at each stage of development in *C. perspicillata*. Box plots capture the variation in the number of PH3-positive cells (**A**) and the size of PH3 signal across all stages (**B**). The total mean number of dividing cells, represented by the dotted horizontal line in **A**, is 1240. The total mean area of PH3 signal is 135.75µm^2^, represented by the dotted horizontal line in **B**. Multiple-pairwise comparisons of the total mean (dotted line) to the mean PH3 value per stage are evaluated with ANOVA. Across development, significant changes in PH3 are observed (p<2.2e-16). The peak number of divisions occur at CS22 and the peak size in cell signal occurs at CS20.


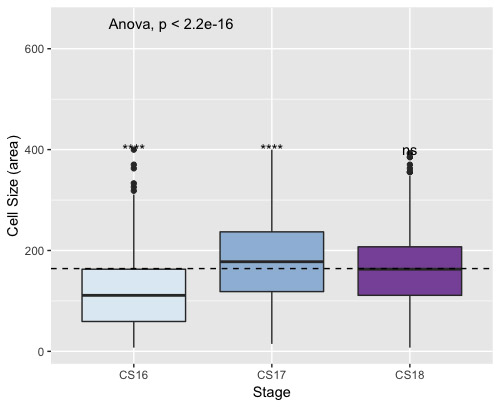


## **Figure S5:**

**Proliferation changes during *Miniopterus* development.** PH3-signal during CS16-CS18 was quantified at each stage of development in *M.natalensis*. Box plots capture the variation in the size of PH3-signal per cell. The mean size of PH3 signal across the three stages of development are represented by the dotted horizontal line. Multiple-pairwise comparisons of the total mean (dotted line) to the mean PH3 value per stage is evaluated with ANOVA. Significant changes in PH3 are observed from the total mean (p<2.2e-16) in CS16 and CS17 but not CS18. The PH3-signal is similar at CS17 and CS18.


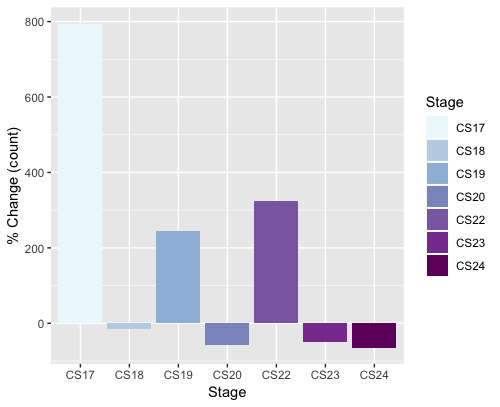


## **Figure S6:**

**Proliferation changes during *Carollia* development.** Percent change in diving cells of model bat *C. perspicillata* from CS16 to CS24. The number of dividing cells was quantified at each stage of development in *C. perspicillata*. The percent change in proliferation at each stage is calculated from the preceding stage. Proliferation is increased from stages CS16-17, CS18-19, and CS20-22. There is similar proliferation at CS17 and CS18 and a slight decrease in proliferation from CS19-20, CS22-23, and CS23-24 (ANOVA, p<2.2e-16). The peak number of divisions occur at CS22 (Figure S4A) and the least number of divisions occur at CS16 (Figure S4A).


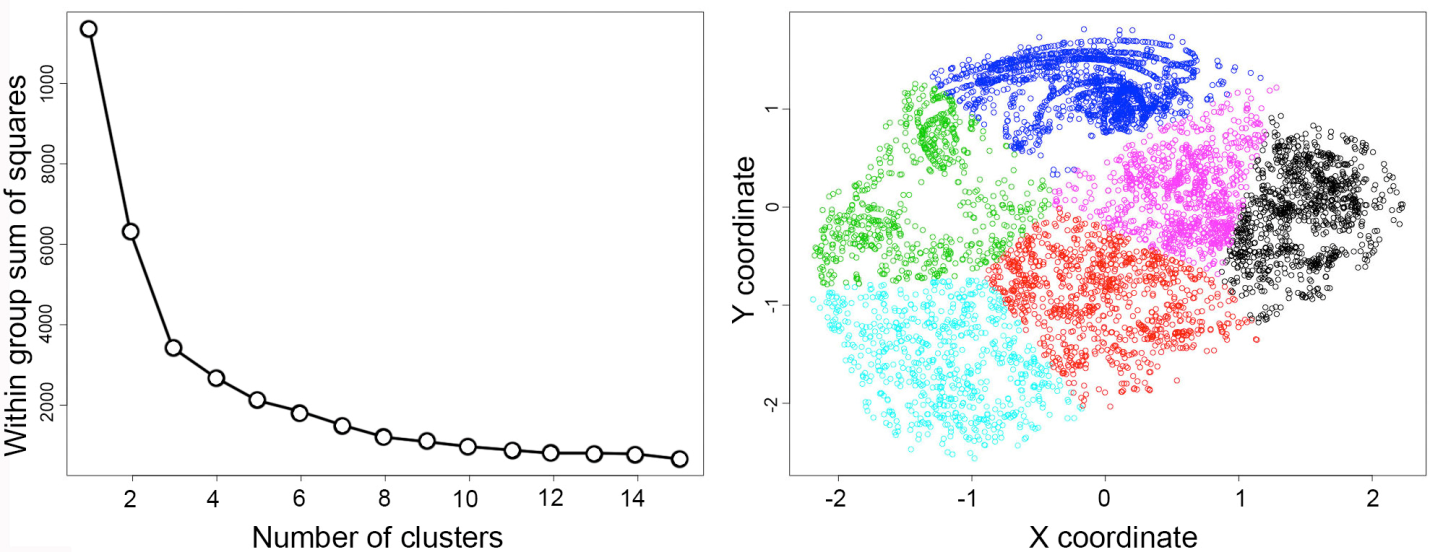


**B**

**A**

## **Figure S7:**

**K-means cluster analysis**. K-means cluster analysis finds groups in data without defined categories, with the number of groups defined by the variable K. The optimal number of clusters was determined with the Elbow method by varying the k-means clustering k-value from 1 to 15 and finding the elbow, or bend, which indicates the most accurate k-value. Within group sum of squares reflect the mean distance between points in a group. K-values 3-6 'bend' the curve (**A**). Visualization of K-means cluster with k=6 highlight group structure (**B**). Distinct developmental regions are highlighted with different colors: anterior midface (black), posterior midface (pink), anterior basicranium (red), posterior basicranium (light blue), hindbrain (green) and forebrain (dark blue). Each cluster defines unique distribution patterns in each region. Proliferation patterns may relate to the concept of modularity known to influence mammalian skull evolution (Goswami 2006).


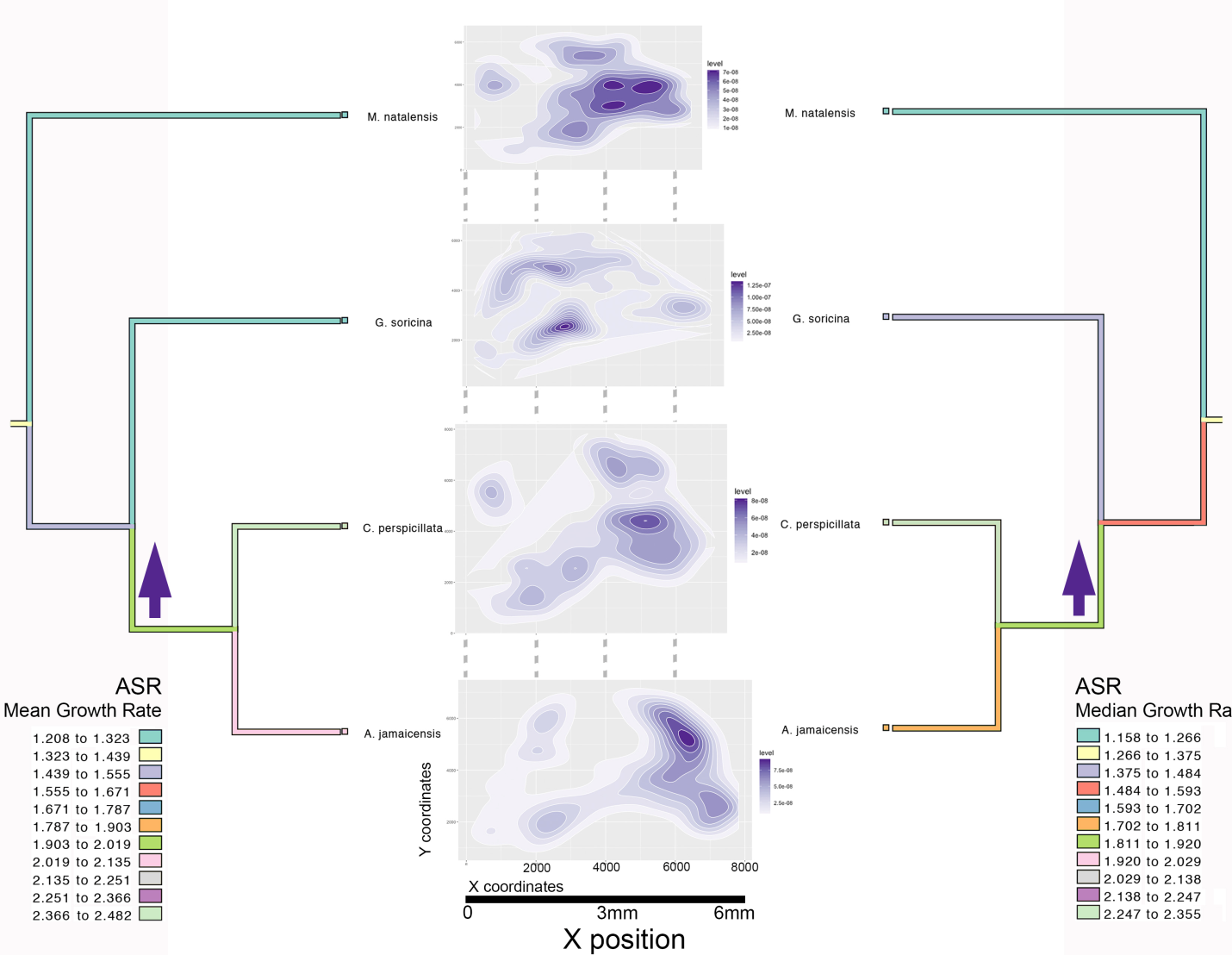


## **Figure S8:**

**PH3 patterns in development and evolution**. Spatial distribution maps of all X,Y positions of PH3-positive cells per species at CS18 are shown on a 2D density map placed alongside the terminal branch of the respective phylogenetic position of each species. All 2D maps are oriented laterally to the same X position, with the midface directed toward the left and the brain orientated dorsally. The topology of the mirror-trees is based on the molecular phylogeny from (Dumont et al. 2014). The phylogeny on the left maps the standardized mean (PH3/DAPI*100) number of proliferating cells in development. The phylogeny on the right maps the standardized median (PH3/DAPI*100) number or proliferating cells. The branches are colored to represent ancestral states based on parsimony reconstruction in Mesquite (Massidon and Maddison 2018) according to the legend. Proliferation is elevated (arrowhead) in the lineage leading to *C. perspicillata* and *A. jamaicensis*. Mean proliferation does not terminally differ between *G. soricina* and *M. natalensis* as a result of parallelism decreasing proliferation within the *G. soricina* lineage.


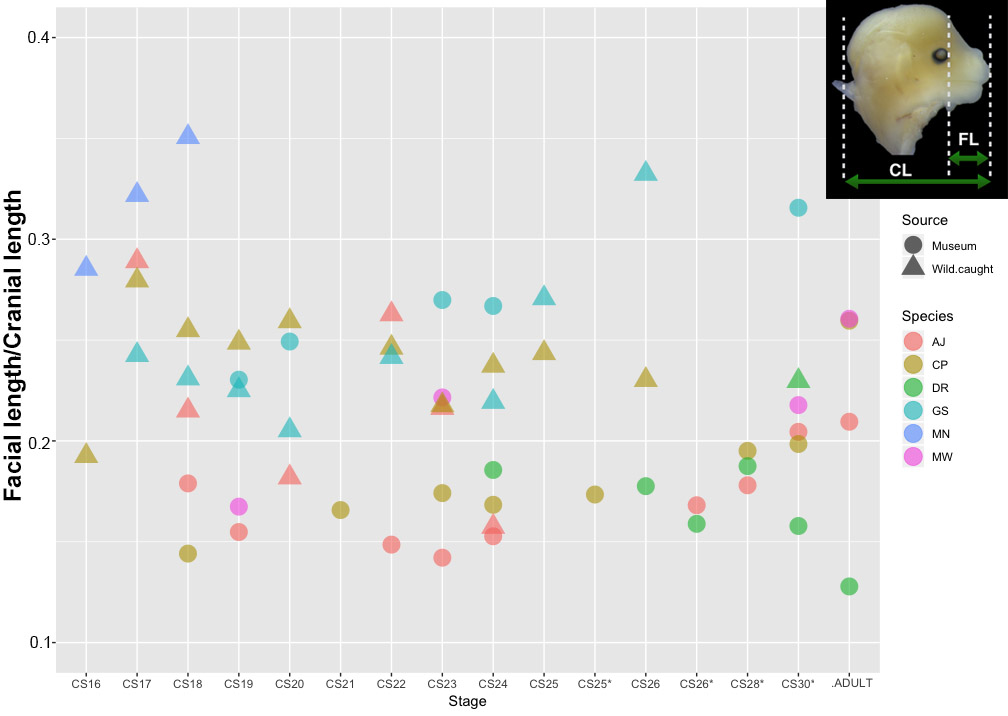


## **Figure S9:**

**Relative facial length development between species**. All bat species are staged with external limb features. To standardize facial length (Table S2) between stages and between species, facial length was divided by cranial length (FL/CL). Field collected and museum collected specimens are shown for six species. From our field studies, only a single species, *C. perspicillata*, had a complete embryonic series (CS16-CS24). *M. natalensis* had three biological replicates for CS16-CS18. *A. jamaicensis* and *G. soricina* had few biological replicates for CS17-CS18 with additional, but sparse samples for CS19-24+ (**Table S1).** Museum specimens ranged from CS18 to adult. FL/CL is shown as an average when n>2. AJ: *A. jamaicensis*. CP: *C. perspicillata*. DR: *Desmodus rotundus*. GS: *G. soricina*. MN: *M natalensis*. MW: *Macrotus waterhousii*.


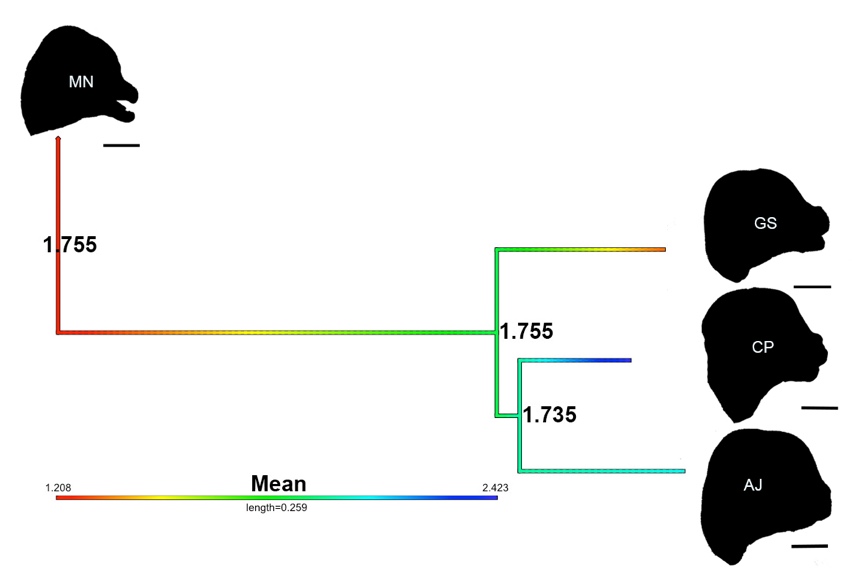


## **Figure S10:**

**Ancestral state reconstruction of proliferation during development**. Standardized mean proliferation (PH3/DAPI*100) was evaluated on a molecular tree and pruned to relevant taxa. Maximum likelihood ancestral state estimates of CS18 proliferation at each node are shown. The evolutionary history is colored along each branch. Ancestral proliferation was compared to modern species proliferation. Along *M. natalensis* (MN) and *G. soricina* (GS), mean proliferation independently decreases from the ancestral mean estimate of 1.755. The mean proliferation increases drastically in *C. perspicillata* (CP) and increases slightly in *A. jamaicensis* (AJ) from an ancestral mean estimate of 1.735. Compared to ancestral proliferation, the growth process in *C. perspicillata* is elevated to 2.423 (p=0.001). In *A. jamaicensis*, compared to ancestral proliferation, the proliferation is slightly elevated to 2.055 (p=0.03).


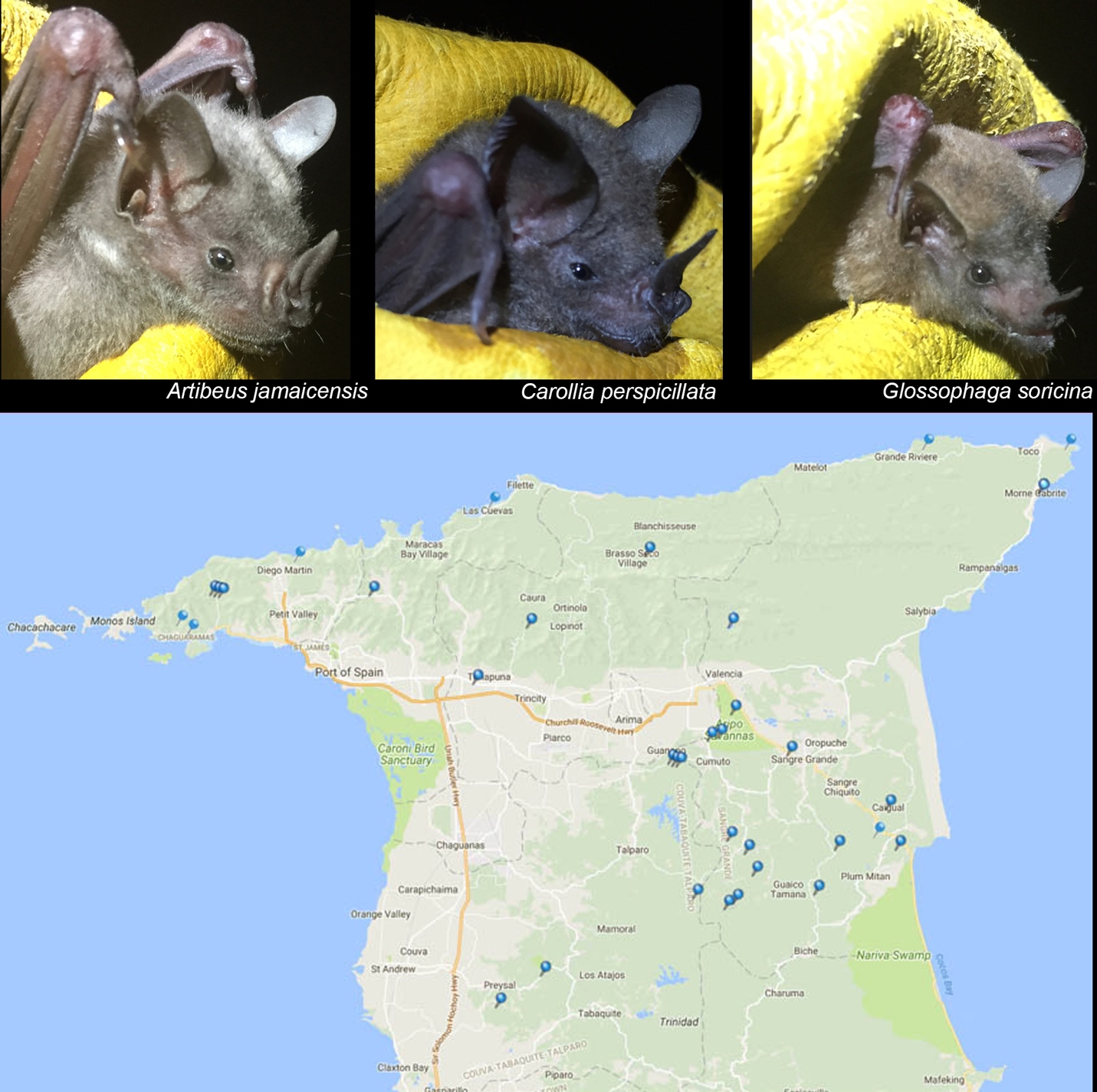


## **Figure S11:**

**Sampling sites in** **Trinidad, West Indies.** Mistnetting and roost locations sampled from 2014-2017. *Artibeus jamaicensis*, *Carollia perspicillata*, and *Glossophaga soricina* were targeted from the continental island of Trinidad. All samples of phyllostomids were from roosts with >200 individuals or from mist-netting. From each roost, a maximum of ten bats per species were removed. Any resampled roost was done with a one-year interval.

## **Figure S12:**

**
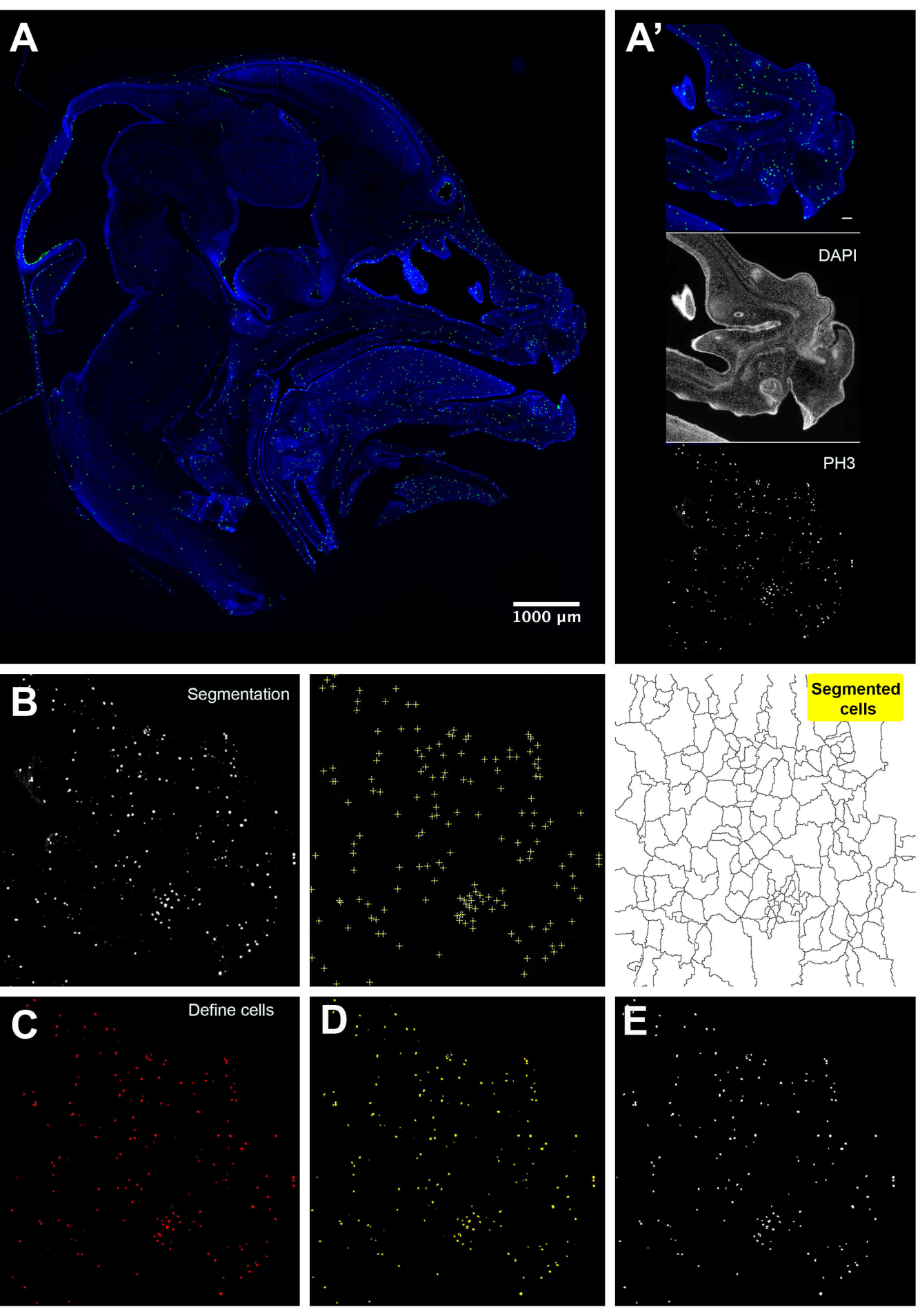
**

## **Figure S12:**

**Segmentation and identification of proliferating cells in bat development.** All cells were automatically identified using a watershed and threshold algorithm. Lateral view of a 10µm-parasagittal section from a CS20 *C. perspicillata* embryo labeled with PH3 (green) and DAPI (blue) (**A**). A small region of the anterior midface (**A’**) is selected to illustrate the methodology. First, the two channels are separated into raw 16-bit TIF images (**A’**). The PH3 channel is selected to illustrate the segmentation and identification process (**B-E**). Individual cells are segmented from the 16-bit TIF with the Find Maxima watershed-based function within FIJI (Schindelin et al. 2012). Cell signal was localized to the nucleus. One local maxima is identified per cell (crosses) and used to generate a new binary image of segmented cells (**B**). To define the area occupied by cells, a threshold was applied on the 16-bit TIF with an Otsu algorithm. After thresholding, a new binary image is created with the area of all the cells defined (**C**). The binary image defining the cells is then combined with the segmented particles with the operation ‘AND’ using Image Calculator in FIJI to create a new binary image with segmented cells, each with a defined area (D). Small (<10 pixels) and large fragments (>600 pixels) are filtered out with Analyze Particles in FIJI to exclude imaging noise. Subsequent analysis is performed on the returned image that contains cells within a biologically relevant size range (**E**). Scale bar in A’: 100µm.

**
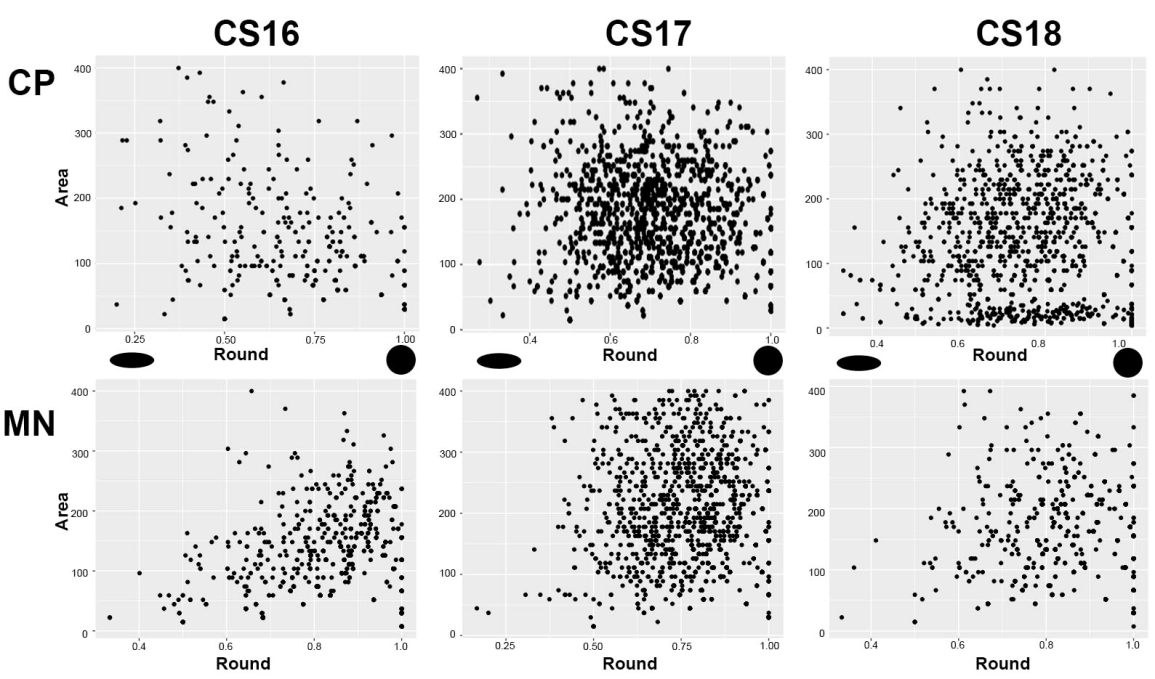
**

**Figure S13:** **Size and shape of PH3 signal.** We investigated size and shape of PH3-positive cells across CS16, CS17 and CS18 for *C. perspicillata* and *M. natalensis*. Size of PH3-signal per cell was evaluated by area. Roundness of PH3-signal per cell was determined by dividing the major axis of the cell body with the minor axis of the cell body. Shape descriptors were investigated to help evaluate cellular behavior (i.e. round cell likely G2 or prophase and elongated cell likely metaphase or telophase). However, we did not find a strong relationship between size and shape.

# **REF**

Behringer R.R., Rasweiler IV J.J., Chen C.H., Cretekos C.J. 2009. Genetic regulation of mammalian diversity. Cold Spring Harb. Symp. Quant. Biol.

Dumont E.R., Samadevam K., Grosse I., Warsi O.M., Baird B., Davalos L.M. 2014. Selection for mechanical advantage underlies multiple cranial optima in new world leaf-nosed bats. Evolution (N. Y). 68:1436–1449.

Goswami A. 2006. Cranial modularity shifts during mammalian evolution. Am. Nat.

Massidon W.P., Maddison D.R. 2018. Mesquite: A modular system for evolutionary analysis. Version 3.51. http://www.mesquiteproject.org.

Schindelin J., Arganda-Carreras I., Frise E., Kaynig V., Longair M., Pietzsch T., Preibisch S., Rueden C., Saalfeld S., Schmid B., Tinevez J.Y., White D.J., Hartenstein V., Eliceiri K., Tomancak P., Cardona A. 2012. Fiji: An open-source platform for biological-image analysis. Nat. Methods. 9:676–682.
