# Supplementary material for: Differential cellular proliferation underlies heterochronic generation of cranial diversity in phyllostomid bats
Source: EvoDevo. 2020 Jun 2;11:11. doi: 10.1186/s13227-020-00156-9 (PMC7268441; doi:10.1186/s13227-020-00156-9)
Supplement: Supplementary file 3 — Additional file 3. Supplemental methods: (1)Sampling, (2) Maximizing experiments with few biological replicates, (3) Automatic cell quantification (4) PH3 as a mitotic marker, (5) Cellular development. [file 13227_2020_156_MOESM3_ESM.docx]

# Sampling

The island of Trinidad was chosen based on its diversity of bats (Goodwin and Greenhall 1961) in a geographically small area (4,769km^2^; Geluso 2019). It has also been a field site for many bat researchers interested in behavior (Kunz and Diaz 1995; Davidson and Wilkinson 2004), physiology (Thomas and Suthers 1972; Heideman et al. 1992; Rasweiler IV and Badwaik 1997; Porter and Wilkinson 2001), and development (Sears et al. 2006; Cretekos et al. 2008). These studies are valuable for documenting the natural history of these bats, especially in regards to reproductive behaviors. Some bats are known to give birth in the rainy season (May - December) and some bats give birth during the dry season (Jan - May). It is easier to capture bats during the dry season, so we focus on common, abundant, and reproductively synchronized species. *Carollia perspicillata*, *Artibeus jamaicensis*, and *Glossophaga soricina* were wild caught in Trinidad during the dry season from February to May (Figure S11).

We expected phyllostomids to have a ~90-120 gestational period based on time-pregnancies in captive colonies (Rasweiler IV 1972; Quintero and Rasweiler IV 1974; Cretekos et al. 2005). We also expected the face to begin midfacial fusion starting at CS15 (~day 46) and complete by CS16 (~day 50) based on what we know from facial morphogenesis in mouse (Losa et al. 2018) and the corresponding morphological events documented in the detailed staging system of *C. perspicillata* (Cretekos et al. 2005). Since we are primarily interested in facial length differences, our goal was to sample bats after fusion of the craniofacial prominences starting at CS16 and subsequently at CS17, CS18, etc, until CS24.

February to March roughly corresponds to CS12-18 although the rainy and dry seasons during 2014-2017 have been variable and unpredictable compared to earlier expeditions (Rasweiler IV et al. 2009), which affected the targeting of embryos. In 2017, hurricanes Harvey, Irma, and Maria affected the export of tissues, delaying the progress of this study.

Sampling sites were varied throughout the year and individual roosts were only sampled once per year. Each artificial roost, like an abandoned water-tank or house, is primarily (~90%) composed of *C. perspicillata*, with ~10% belonging to *G. soricina*. In artificial roosts of ~90% *G. soricina*, ~10% belong to *Saccopteryx bilineata*. Artificial *A. jamaicensis* roosts did not contain additional species cohabiting the same structure, but *Phyllostomus discolor* with pups were found to roost in nearby structures. Some structures contained *C. perspicillata*, *G. soricina*, and *Phyllostomus hastatus* with newborns roosting at different levels. When sampling from roosts, we used modified butterfly nets to gently capture bats as they roosted.

For collection by mist-netting, we deployed bat-friendly nets ranging from 2.6m and up to 18m (Avinet, Inc.) at ground level along forest trails, over water, in cocoa and banana plantations, and ranches with owner permission. In open field or large trails with high tree canopy, we used a triple-high net system with 9m to 18m nets. We set up mist-nets before dusk, opened nets at sunset, and closed nets after 4.5 hours. Nets were monitored every 10 minutes during peak bat activity periods, from 6PM to 7PM, then every 20 minutes. Outside caves, we set up harp traps at dusk. The harp trap was constantly monitored by up to four people from dusk until 10PM. We identified bats by species and held pregnant females in cloth bags for up to an hour until subsequent screening. For pregnant females, length of forearm, body weight, and reproductive status (early, mid-, or late pregnant) were recorded.

Up to ten pregnant females were sampled per roost of 200 individuals or per mist-netting site. Of the pregnant females, each was evaluated for embryo age based on size approximations from gentle abdominal palpation. The size of CS14 in *C. perspicillata* is 6.9-7mm (Cretekos et al. 2005) and the long axis of the uterus is about 9-10mm (unpublished observation). Thus, sizes less than 8mm were excluded due to unreliability of gestational age based on abdominal palpation.

All embryonic bats that were collected from the wild in Trinidad, West Indies are shown in Table S1 and staged according to limb morphology. *Carollia perspicillata* had a complete range of embryonic stages (CS16-CS24) with biological replicates (n=3 per stage). *Miniopterus natalensis* had a subset of embryonic ages (CS16-CS18) with biological replicates (n=3 per stage). *Artibeus jamaicensis* and *Glossophaga soricina* had a range of embryonic stages (CS17-CS24) with few biological replicates (n=1-2). Adult female specimens for each embryo were processed for acquisition into the Museum of Comparative Zoology at Harvard when possible. The majority of bats used for this study were obtained through a collaboration with Dr. Janine Seetahal for her dissertation research on rabies and other viruses (Malmlov et al. 2015, 2017; Seetahal et al. 2018, 2019), requiring brain tissues from adults.

All of our interpretations of data were based on prior knowledge about facial development from mouse studies. We initially expected the CS15 bat to match to mouse E10.5. We noted however, that we needed to compare mice starting at E14.5 to match facial development to the bat at CS16 (~day 50; Figure S1).

# Maximizing experiments with few biological replicates

Cranial tissue from each species of bat through craniofacial development from CS16-CS24 was processed for cryosectioning. Since sample size was limited, we decided to use serial sections to capture the anterior-posterior length of the head. The ideal orientation to capture the length along the anterior-posterior axis was sagittal. Each 10µm-sagittal section spanning the medial-lateral aspects of the head (Figure 3) was collected on a series of 10 slides. Each slide series had one section collected for every 100µm. One series was stained for histological reference for acquisition into the Museum of Comparative Zoology at Harvard and another series was used to detect proliferation with immunohistochemistry. All additional slide series were stored at -80°C.

# Automatic cell quantification

Recent improvements in automatic, high-throughput, high-resolution imaging (Ghaznavi et al. 2013; Fu et al. 2017), and automated imaging analysis (Schindelin et al. 2012; Yan and Verbeek 2012) means cells from a wide range of tissues and species can be studied in detail. Numerous metrics can be extracted from image data with relatively little effort with use of an algorithm, like the National Institute of Health’s ImageJ (Schindelin et al. 2012), to better characterize cells. This task represents a time-consuming process to segment, threshold, and measure features of cells per channel, per section, per specimen. For example, specimens in this study are represented by at least 10 sections, with each section composed of multiple images stitched together to capture the entire sagittal view of the head, which ranges in size from 4.3mm in CS16 to 14mm at CS24 in *C. perspicillata*. The multiple, large field of view makes individual user adjusted analysis of each image labor intensive, subjective, and time consuming. Automation was necessary to systematically measure large number of cells from large fields of view.

To address this, we developed scripts to automate the built-in image analysis within ImageJ. The general outline of our algorithm to analyze cells is shown in Figure S12. We wrote scripts in java to automate these user tasks within ImageJ. Because we did not know the exact role cellular proliferation would have during craniofacial development, we data-mined as many metrics as possible: cell counts, size, shape, coordinates, and pixel intensity per cell. A single script was used for all species and stages of craniofacial development. All immunohistochemistry experiments and imaging settings were similar, a necessary prerequisite to automated image analysis.

# PH3 as a mitotic marker

After immunohistochemistry targeting of PH3, all serial sections were imaged with an Olympus VS120 slide scanner at 20x and aligned by two Procrustes transformations, translation and rotation, with the FIJI Plugin *StackReg*. Aligned images were individually processed to identify PH3-signal (Figure S12). PH3-signal information (position, size, shape) were collected from each section. Position is the X, Y coordinates of each cell maxima. Size is presented as the area of signal per cell. Shape is described as roundness and determined by dividing the major axis of the cell body with the minor axis of the cell body.

At CS16, the median cell area (Figure 4) labeled with PH3 is 66.64µm^2^ (cells measured (n) = 634). At CS17 (n=5674), this increase by 88% to a median cell area of 125.87µm^2^, which then slightly decreases to 111.07µm^2^ at CS18 (n=4878) and CS19 (n=16765). A 47% increase in the median area, 162.89µm^2^ (n=7155), is observed at CS20. At CS22, the size of PH3 signal decreases by 24% to 123.45µm^2^ (n=30274) and at CS23, the size decreases by 12% (n=15651). By stage CS24, PH3 signal per cell decreases by 81% to 26µm^2^ (n=5553). In *M. natalensis*, a similar size range in PH3 expression is observed (Figure S5) during craniofacial development: at CS16, cell size is 111.05µm^2^ (n=1013), which increases by 60% to 177.7µm^2^ (n=3194) at CS17, and then slightly decreases to 162.89µm^2^ at CS18 (n=1432).

The range of size in PH3-signal is related to the phase of the cell cycle (GURLEY et al. 1978). We noted that size of PH3-signal in *C. perspicillata* also relates to the phase, or shape, of the cell in mitosis (Figure S2). However, when we regress PH3-signal size to the shape of the cell (Figure S13), a weak pattern is observed. In *C. perspicillata*, larger cells are rounder in CS16 (R-square=0.1885, p<0.001) and CS17 (R-square=0.0281, p<0.001). This is possibly related to cells in metaphase of mitosis. In *M. natalensis*, a similar pattern is found at CS16 (R-square=0.0667, p<0.001). Overall, size and shape do not show a strong relationship.

The weak coefficient of determination between size and shape may reflect the heterogeneous composition of cells, different progenitor populations, or the dynamic nature of cell shape during mitosis. For example, we know from the basicranium, dividing cartilage cells in the growth plate can rapidly switch from flat morphology to rounded morphology by swelling in size during terminal divisions (Farnum et al. 2002; McBratney-Owen et al. 2008; Whitaker et al. 2017). How cell size relates to morphological variation of the cell during mitosis merits further investigation.

We quantify and compare the number of PH3-positive cells and the PH3 area per cell across all stages in *C. perspicillata*. The mean number and range of proliferating cells per stage are shown as boxplots in Figure S4A. The mean and range of PH3 area per cell, per stage is shown in Figure S4B. We present the difference as the percentage change in Figure S3 and Figure S6 to highlight the robust increases in proliferation number and both increases and decreases in median size as development progresses.

We quantify and compare the amount PH3-signal across all stages in *M. natalensis*. The mean and range of PH3 area per cell per stage is shown in Figure S5. Significant changes in PH3-signal size are observed at CS16 and CS17. At CS16, cell-size is decreased, possibly relating to a lesser proportion of cells in metaphase. At CS17, PH3-signal size is elevated, possibly relating to an increase in the proportion of cells in metaphase. The change in cell size from CS16-CS18 is similar in *C. perspicillata*.

# Cellular development

The head is a complex structure composed of multiple parts derived from different tissues. We examine proliferation across the entire craniofacial complex and propose that modular changes in tissue growth (i.e. proliferation) facilitate morphological evolution in phyllostomid bats, including novel and exaggerated forms. The extracted positional information (X, Y) of detected PH3-cell maxima across all serial sections was used to generate a point pattern in R. The spatial distribution of proliferating cells is represented as density data on 2D contour plots, where dark regions relate to concentrated cells and lighter regions are less dense. The 2D contour plots provide quantitative information on the distribution of PH3-signal. For comparisons through development, the 2D contour plots highlight spatiotemporal similarities and differences of growth.

We quantify and compare the normalized PH3-signal between species to account for size differences during development. To standardize, the total amount of PH3-positive cells was divided by the total amount of DAPI-positive cells and multiplied by 100 (percent area fraction). Group mean and median proliferation at CS18 (n=3 per species) were mapped onto the tips of the phylogeny shown in Figure S8, S10 to document the evolution of development. Along *M. natalensis* and *G. soricina*, mean proliferation independently decreases from the ancestral mean estimate of 1.755. The mean proliferation increases drastically in *C. perspicillata* and increases slightly in *A. jamaicensis* from an ancestral mean estimate of 1.735. Spatial distribution maps are placed alongside respective phylogenetic position to highlight where proliferation differences are occurring between phyllostomid species (Figure S8). We observe that proliferation in the midface is elevated in the lineage leading to *C. perspicillata* and *A. jamaicensis*. Mean proliferation does not terminally differ between *G. soricina* *and M. natalensis*.

References

Cretekos C.J., Wang Y., Green E.D., Martin J.F., Rasweiler IV J.J., Behringer R.R. 2008. Regulatory divergence modifies limb length between mammals. Genes Dev. 22:141–151.

Cretekos C.J., Weatherbee S.D., Chen C.H., Badwaik N.K., Niswander L., Behringer R.R., Rasweiler IV J.J. 2005. Embryonic staging system for the short-tailed fruit bat, Carollia perspicillata, a model organism for the mammalian order Chiroptera, based upon timed pregnancies in captive-bred animals. Dev. Dyn. 233:721–738.

Davidson S.M., Wilkinson G.S. 2004. Function of male song in the greater white-lined bat, Saccopteryx bilineata. Anim. Behav.

Farnum C.E., Lee R., O’Hara K., Urban J.P.G. 2002. Volume increase in growth plate chondrocytes during hypertrophy: The contribution of organic osmolytes. Bone.

Fu X., Lennerz J.K., Onozato M., Iafrate A., Yagi Y. 2017. Evaluation of a confocal WSI scanner for FISH slide imaging and image analysis. Diagn. Pathol. 3.

Geluso K. 2019. A survey of bats in northern Trinidad late in the rainy season /. .

Ghaznavi F., Evans A., Madabhushi A., Feldman M. 2013. Digital Imaging in Pathology: Whole-Slide Imaging and Beyond. Annu. Rev. Pathol. Mech. Dis.

Goodwin G.G., Greenhall A.M. 1961. A Review of the Bats of Trinidad and Tobago. Bull. Am. Museum Nat. Hist. 122:187–302.

GURLEY L.R., D’ANNA J.A., BARHAM S.S., DEAVEN L.L., TOBEY R.A. 1978. Histone Phosphorylation and Chromatin Structure during Mitosis in Chinese Hamster Cells. Eur. J. Biochem.

Heideman P.D., Deoraj P., Bronson F.H. 1992. Seasonal reproduction of a tropical bat, Anoura geoffroyi, in relation to photoperiod. J. Reprod. Fertil.

Kunz T.H., Diaz C.A. 1995. Folivory in Fruit-Eating Bats, with New Evidence from Artibeus jamaicensis (Chiroptera: Phyllostomidae). Biotropica. 27:106.

Losa M., Risolino M., Li B., Hart J., Quintana L., Grishina I., Yang H., Choi I.F., Lewicki P., Khan S., Aho R., Feenstra J., Vincent C.T., Brown A.M.C., Ferretti E., Williams T., Selleri L. 2018. Face morphogenesis is promoted by Pbx-dependent EMT via regulation of snail1 during frontonasal prominence fusion. Dev.

Malmlov A., Seetahal J., Carrington C., Ramkisson V., Foster J., Miazgowicz K.L., Quackenbush S., Rovnak J., Negrete O., Munster V., Schountz T. 2017. Serological evidence of arenavirus circulation among fruit bats in Trinidad. PLoS One.

Malmlov A., Seetahal J., Carrington C., Ramkisson V., Foster J., Munster V., Quackenbush S., Schountz T. 2015. Serological evidence that Tacaribe virus is circulating among bats in Trinidad and Tobago. Eur. J. Mol. Clin. Med.

McBratney-Owen B., Iseki S., Bamforth S.D., Olsen B.R., Morriss-Kay G.M. 2008. Development and tissue origins of the mammalian cranial base. Dev. Biol. 322:121–132.

Porter T. a., Wilkinson G.S. 2001. Birth synchrony in greater spear-nosed bats (Phyllostomus hastatus). J. Zool. 253:383–390.

Quintero F., Rasweiler IV J.J. 1974. Ovulation and early embryonic development in the captive vampire bat, Desmodus rotundus. J. Reprod. Fertil.

Rasweiler IV J.J. 1972. Reproduction in the long-tongued bat, Glossophaga soricina. I. Preimplantation development and histology of the oviduct. J. Reprod. Fertil.

Rasweiler IV J.J., Badwaik N.K. 1997. Delayed development in the short-tailed fruit bat, Carollia perspicillata. J. Reprod. Fertil. 109:7–20.

Rasweiler IV J.J., Cretekos C.J., Behringer R.R. 2009. Collection of embryos from short-tailed fruit bats (Carollia perspicillata). Cold Spring Harb. Protoc. 2009:pdb--prot5162.

Schindelin J., Arganda-Carreras I., Frise E., Kaynig V., Longair M., Pietzsch T., Preibisch S., Rueden C., Saalfeld S., Schmid B., Tinevez J.Y., White D.J., Hartenstein V., Eliceiri K., Tomancak P., Cardona A. 2012. Fiji: An open-source platform for biological-image analysis. Nat. Methods. 9:676–682.

Sears K.E., Behringer R.R., Rasweiler IV J.J., Niswander L.A. 2006. Development of bat flight: morphologic and molecular evolution of bat wing digits. Proc. Natl. Acad. Sci. U. S. A. 103:6581–6.

Seetahal J.F.R., Sanchez-Vazquez M.J., Vokaty A., Carrington C.V.F., Mahabir R., Adesiyun A.A., Rupprecht C.E. 2019. Of bats and livestock: The epidemiology of rabies in Trinidad, West Indies. Vet. Microbiol.

Seetahal J.F.R., Vokaty A., Vigilato M.A.N., Carrington C.V.F., Pradel J., Louison B., Van Sauers A., Roopnarine R., González Arrebato J.C., Millien M.F., James C., Rupprecht C.E. 2018. Rabies in the Caribbean: A situational analysis and historic review. Trop. Med. Infect. Dis.

Thomas S.P., Suthers R.A. 1972. The Physiology and Energetics of Bat Flight. J. Exp. Biol. 57:317–335.

Whitaker A.T., Berthet E., Cantu A., Laird D.J., Alliston T. 2017. Smad4 regulates growth plate matrix production and chondrocyte polarity. Biol. Open.

Yan K., Verbeek F.J. 2012. Segmentation for high-throughput image analysis: Watershed masked clustering. Lect. Notes Comput. Sci. (including Subser. Lect. Notes Artif. Intell. Lect. Notes Bioinformatics).
